# Dual-function antimicrobial-antibiofilm peptide hybrid to tackle biofilm-forming *Staphylococcus epidermidis*

**DOI:** 10.1186/s12941-024-00701-7

**Published:** 2024-05-16

**Authors:** Mathira Wongchai, Saharut Wongkaewkhiaw, Sakawrat Kanthawong, Sittiruk Roytrakul, Ratchaneewan Aunpad

**Affiliations:** 1 Graduate Program in Biomedical Sciences, Faculty of Allied Health Sciences, Thammasat University, Pathum Thani, Thailand; 2 School of Dentistry, King Mongkut’s Institute of Technology Ladkrabang, Bangkok, Thailand; 3 Department of Microbiology, Faculty of Medicine, Khon Kaen University, Khon Kaen, Thailand; 4 Functional Proteomics Technology Laboratory, National Center for Genetic Engineering and Biotechnology, National Science and Technology Development Agency, Pathum Thani, Thailand

**Keywords:** Antibiofilm peptide, *Staphylococcus epidermidis*, Biofilm, Antibacterial activity, Lipid binding motif

## Abstract

**Background:**

Due to their resistance and difficulty in treatment, biofilm-associated infections are problematic among hospitalized patients globally and account for 60% of all bacterial infections in humans. Antibiofilm peptides have recently emerged as an alternative treatment since they can be effectively designed and exert a different mode of biofilm inhibition and eradication.

**Methods:**

A novel antibiofilm peptide, BiF, was designed from the conserved sequence of 18 α-helical antibiofilm peptides by template-assisted technique and its activity was improved by hybridization with a lipid binding motif (KILRR). Novel antibiofilm peptide derivatives were modified by substituting hydrophobic amino acids at positions 5 or 7, and both, with positively charged lysines (L5K, L7K). These peptide derivatives were tested for antibiofilm and antimicrobial activities against biofilm-forming *Staphylococcus epidermidis* and multiple other microbes using crystal violet and broth microdilution assays, respectively. To assess their impact on mammalian cells, the toxicity of peptides was determined through hemolysis and cytotoxicity assays. The stability of candidate peptide, BiF2_5K7K, was assessed in human serum and its secondary structure in bacterial membrane-like environments was analyzed using circular dichroism. The action of BiF2_5K7K on planktonic *S. epidermidis* and its effect on biofilm cell viability were assessed via viable counting assays. Its biofilm inhibition mechanism was investigated through confocal laser scanning microscopy and transcription analysis. Additionally, its ability to eradicate mature biofilms was examined using colony counting. Finally, a preliminary evaluation involved coating a catheter with BiF2_5K7K to assess its preventive efficacy against *S. epidermidis* biofilm formation on the catheter and its surrounding area.

**Results:**

BiF2_5K7K, the modified antibiofilm peptide, exhibited dose-dependent antibiofilm activity against *S. epidermidis*. It inhibited biofilm formation at subinhibitory concentrations by altering *S. epidermidis* extracellular polysaccharide production and quorum-sensing gene expression. Additionally, it exhibited broad-spectrum antimicrobial activity and no significant hemolysis or toxicity against mammalian cell lines was observed. Its activity is retained when exposed to human serum. In bacterial membrane-like environments, this peptide formed an α-helix amphipathic structure. Within 4 h, a reduction in the number of *S. epidermidis* colonies was observed, demonstrating the fast action of this peptide. As a preliminary test, a BiF2_5K7K-coated catheter was able to prevent the development of *S. epidermidis* biofilm both on the catheter surface and in its surrounding area.

**Conclusions:**

Due to the safety and effectiveness of BiF2_5K7K, we suggest that this peptide be further developed to combat biofilm infections, particularly those of biofilm-forming *S. epidermidis*.

**Supplementary Information:**

The online version contains supplementary material available at 10.1186/s12941-024-00701-7.

## Background

Hospital-associated infections (HAIs), also referred to nosocomial infections, pose a significant global public health concern. Each year, there were around 40,000 hospitalized patients’ death due to these infections and an escalating mortality was observed both in developing and developed countries [1, 2]. More than half (50–70%) of HAIs are caused by the development of biofilms on medical devices which are increasingly used in many aspects of patient care [2–4]. In healthcare settings, the presence of biofilm-related infections is significant concern since they can compromise the effectiveness of antibiotic and immune therapeutics [5]. Thereby, they contribute to the problem of antibiotic resistance and the persistence of infections [6].

Infections caused by *Staphylococcus epidermidis* are a significant global concern, as they account for approximately 80% of infections associated with medical devices [3]. Normally, *S. epidermidis* is considered a non-pathogenic commensal member of human skin flora, colonizing the skin with relatively non-invasive mechanisms, and expressing few virulence factors. However, it has a remarkable capability to attach to and colonize the surfaces of medical devices, forming biofilms which are associated with subsequent infections [7]. The distinctive features characterizing *S. epidermidis* biofilms include the production of polysaccharide intracellular adhesin (PIA) by the intracellular adhesion (*ica*) gene locus, and biofilm regulatory control through the accessory gene regulator (*agr*) quorum-sensing system which is conserved across various staphylococcal species [8].

The treatment of biofilm infections is often challenging, and antibiotic combinations are typically recommended. However, higher concentrations of each antibiotic than against planktonic cells are generally required [9]. Regrettably, eradication of device-related infections typically requires removal of the device and there is a high risk of reinfection, along with patient trauma and additional cost [4]. For these reasons, there is necessary to develop novel antibiofilm agents as well as improved strategies to treat biofilm-associated infections.

Antibiofilm peptides provide a promising approach for combating biofilm-causing bacterial infections, with need particularly focused on infections of indwelling medical devices. One noteworthy feature of antibiofilm peptides is their versatility in regard to targeting of bacteria, whether planktonic (free-floating) or embedded within biofilms [10]. Moreover, a significant advantage of peptides is their low propensity to induce resistance at subinhibitory concentrations, likely due to their mechanistic differences from conventional antibiotics [11, 12]. These peptides exert their antibiofilm effects through diverse mechanisms which vary with the concentration of the peptide. At inhibitory concentrations or higher, peptides possess the capacity to directly kill bacteria before biofilm formation, within the biofilm matrix, or during the detachment process. At subinhibitory concentrations, the peptides exhibit distinct mechanisms which include interference with gene expression, bacterial adhesion, or host immune response [10, 13]. This variety of mechanisms makes these peptides highly effective against bacterial biofilms at various stages of development.

Rational design of antimicrobial peptides using sequence optimization and modification has been widely recognized since it can significantly enhance effectiveness, stability, and specificity [14]. In this study, various strategies, including peptide hybridization and amino acid substitution, were exploited to optimize design of the antibiofilm peptide. The hybrid peptide R-FV-I16, with robust antimicrobial and antibiofilm activities, was obtained via the insertion of a non-functional sequence (RR7) along with an antibiofilm sequence (FV7) [15]. The substitution of leucine and lysine in Temporin 1Tb, yielding the peptide TB_L1FK, has demonstrated an increase in antimicrobial activity and concurrent inhibition of biofilm formation [16].

In this research, a novel and highly effective antibiofilm peptide was designed by combining various strategies including a template-assisted approach, peptide hybridization and sequence modification. A conserved sequence derived from a well-established 18 α-helical antibiofilm peptide was hybridized with a previously described lipid-binding motif, the KILRR motif [17]. The inclusion of a lipid-binding motif represents an intriguing strategy for increasing the peptide’s affinity for bacterial membrane and lipid constituents within the biofilm matrix. This novel peptide serves as a basic scaffold for the development of a series of derivative peptides by amino acid substitution. Among them, BiF2_5K7K, a lysine-substituted hybrid peptide, exhibited remarkable antibiofilm and antimicrobial activities, particularly against *S. epidermidis* biofilm-forming strains, with minimal hemolytic effect and cytotoxicity. Even at subinhibitory concentrations, BiF2_5K7K was able to prevent the formation of *S. epidermidis* biofilm via modulating biofilm-related genes expression. Additionally, BiF2_5K7K demonstrated its ability to prevent biofilm formation on catheter surfaces. This work marked a significant step toward the development of novel antibiofilm agents, which may have implications for combating biofilm infections, particularly those associated with medical devices.

## Materials and methods

### Bacterial strains and growth conditions

The biofilm-forming bacteria, including *S. epidermidis* ATCC 35984, *Staphylococcus aureus* ATCC 25923, *Bacillus cereus* ATCC 11778, *Listeria monocytogenes* 10403s, *Pseudomonas aeruginosa* ATCC 27853, *Salmonella* Typhimurium ATCC 13311, *Escherichia coli* ATCC 25922 and *Acinetobacter baumanii* MT strain, were used. All bacterial strains were grown on tryptic soy agar (TSA; BD Bacto™, USA) except *E. coli* and *L. monocytogenes* which were cultured on Luria agar (LA) and TSA supplemented with 0.6% Yeast Extract (TSAYE), respectively, at 37°C overnight. Subsequently, isolated colonies were inoculated and cultured in 5 mL of tryptic soy broth (TSB; BD Bacto™, USA), Luria Broth (LB), or TSB supplemented with 0.6% Yeast Extract (TSBYE) at 37°C for 16–18 h.

### Peptide design and *in silico* characteristics

Using a lesson learned from peptides designed by a template-assisted approach, a novel antibiofilm peptide, BiF, was developed from the alignment of conserved sequences of 18 α-helical antibiofilm peptides obtained from APD3 (Antimicrobial Peptide Database, retrieved on 7 August 2019). To further enhance its antibiofilm activity, a lipid binding motif (or KILRR motif [17]) was hybridized at the C-terminus of BiF and transformed into hybrid BiF2 peptide. In accord with the results of helical wheel projections and 3D structures, BiF2 was subsequently modified by substituting in one and two hydrophobic amino acids at positions 5 and 7 with lysines (L5K, L7K), positively charged amino acid, to obtain two hybrid derivatives, BiF2_5K and BiF2_5K7K, respectively. Peptide sequences were *in silico* analysed by APD3: Antimicrobial Peptide Calculator and Predictor (https://aps.unmc.edu/prediction). Both hydrophobicity (<H>) and hydrophobic moment (<μH>), as well as helical wheel projection, were determined using the HeliQuest web server (https://heliquest.ipmc.cnrs.fr/). Moreover, I-TASSER (https://zhanglab.dcmb.med.umich.edu/I-TASSER/) was employed to predict the 3D structure of these peptides.

### Peptide synthesis and preparation

The peptides were generated using solid-phase peptide synthesis with 9-fluorenylmethoxycarbonyl (Fmoc) as trifluoroacetate salts and amidation at the C-terminus (China Peptides, China). Reverse phase high-performance liquid chromatography (RP-HPLC; >98% purity) and mass spectrometry were employed to confirm the identity and purity of the peptide sequences, respectively. The peptides were dissolved in sterile deionized water and kept at -20°C until used.

### Biofilm formation capacity of *S. epidermidis* ATCC 35984

The capability of *S. epidermidis* ATCC 35984 to form biofilm was evaluated by crystal violet (CV) staining as described previously [18]. Briefly, bacterial suspensions derived from an overnight culture were harvested and centrifuged at 2,500 × g for 5 min to collect the pellet. Then, TSB was used to resuspended and diluted the pellet to achieve a concentration of 10^6^ CFU/mL at optimal density (OD) of 620 at 0.05. These diluted suspensions were subsequently dispensed into 96-well plates and statically incubated for 24 h at 37°C. Following the incubation period, both the culture media and planktonic cells were discarded, and the biofilm in each well was washed thrice with 0.85% NaCl. Subsequently, the biofilms were fixed using 95% ethanol and stained with a 0.1% (v/v) solution of CV solution (Sigma-Aldrich, USA) for 15 min. Excess dye was removed, and each well was washed thrice with 0.85% NaCl before resuspended in 95% ethanol to dissolve the bound dye. Absorbance was determined at 570 nm using a Multiskan SkyHigh microplate reader (Thermo Scientific™, Singapore). Wells containing only TSB medium without bacteria served as the negative control. All experiments were conducted with six technical replicates and repeated independently three times. The biofilm-forming ability of the bacteria was categorized into four groups based on the OD570 value: no biofilm production (OD570≤ OD_c_), weak biofilm production (OD_c_<OD570≤ 2 × OD_c_), moderate biofilm production (2 × OD_c_<OD570≤4 × OD_c_), and strong biofilm production (4 × OD_c_<OD570). The mean OD ± three standard deviations (SD) of the OD value of the negative control was defined as cut-off OD (OD_c_).

### Biofilm-formation inhibitory activity

The biofilm inhibitory activity of peptide derivatives was assessed by modified crystal violet (CV) staining [19]. Briefly, the pellet of *S. epidermidis* (ATCC 35984) was collected through centrifugation of bacterial suspensions grown overnight at 2,500 × g for 5 min. Subsequently, the pellet was resuspended and diluted with TSB to a final concentration of 10^6^ CFU/mL. Then, 50 µL of these diluted suspensions was transferred to 96-well microplates containing same volume of peptide derivatives in 10 mM phosphate buffer saline (PBS; Caisson Labs, USA), with concentrations ranging from 0.98 to 250 µg/mL. Vancomycin was used as positive control while TSB medium without bacteria was used as negative control. After 24 h of incubation and reaching static growth at 37°C, culture media as well as planktonic cells were removed, then the biofilm in each well was washed thrice with 0.85% NaCl. For CV staining, biofilms were fixed with 95% ethanol and stained with a 0.1% (v/v) CV solution for 15 min. After removal of excess dye, each well was washed thrice with 0.85% NaCl and resuspended in 95% ethanol to dissolve bound dye. Absorbance was determined at 570 nm using a Multiskan SkyHigh microplate reader (Thermo Scientific™, Singapore). All experiments were done in six technical replicates and repeated independently three times. Minimum biofilm inhibitory concentrations at 50% and 90% (MBIC_50_ and MBIC_90_) refers to concentrations of the peptide at which the inhibition of biofilm was at least 50% and 90%, respectively [19, 20].

### Antimicrobial activity

The minimal inhibitory concentrations (MICs) of peptide derivatives versus planktonic bacteria were assessed using a modification of the broth microdilution assay previously described in the National Committee for Clinical Laboratory Standards (NCCLS) [21]. In brief, bacteria at mid-logarithmic growth were collected by centrifugation at 2,500 × g for 5 min. Subsequently, the pellet was resuspended and adjusted in Mueller-Hinton Broth (MHB; BD Bacto™, USA) to an OD620 of 0.05. Fifty µL of bacterial suspensions was transferred to 96-well microplates. Later, 50 µL of peptide derivatives at 0.98–250 µg/mL in 10 mM PBS, was added to wells and incubated for 24 h at 37°C with continuous shaking at 220 rpm. After incubation, the minimum concentration of peptide that resulted in no bacterial growth observed by visible inspection after 24 h of incubation was defined as the MIC [19]. The experiments were done in three technical replicates and repeated independently.

To determine minimal bactericidal concentrations (MBCs) of the peptides, 50 µL of supernatant from non-turbid wells was plated on TSA, and the number of colony-forming units (CFUs) was observed after 24 h of incubation at 37°C. MBC was defined as the minimum concentration of peptide at which no bacterial colony was observed on the plate [19].

### Hemolysis activity

The effect of peptides on human red blood cells (hRBCs) was assessed by observing the release of hemoglobin upon treated with peptide [16]. Briefly, the hRBCs were obtained from healthy volunteers and subsequently diluted in 10 mM PBS to a concentration of 2% hRBCs. An equal volume of 2% hRBCs and peptide at concentration of 0.98–250 µg/mL in 10 mM PBS mixtures were incubated for 1 h at 37°C. Following incubation, the suspension was centrifuged, then supernatants were collected and transferred to 96-well microplates for measurement of absorbance at OD of 405 nm (OD405). This study was performed in accordance with the Thammasat University Ethics Committee (COA No. 066/2562). Written informed consent was obtained from all volunteers.

### Biofilm cell viability

The biofilm cell viability after treated with hybrid peptide BiF2_5K7K was examined by 3-(4,5-Dimethylthiazol-2-yl)-2,5-Diphenyltetrazolium bromide or MTT colorimetric assays [16]. Bacterial suspensions (10^6^ CFU/mL in TSB) and peptide at concentration ranging from 0.98 to 250 µg/mL in 10 mM PBS were incubated in 96-well microtiter plates at 37°C for 24 h. After incubation, supernatant was discarded, followed by the addition of 0.4 mg/mL MTT (Sigma-Aldrich, USA) in 10 mM PBS to each well and incubated for 3 h at 37°C. Subsequently, supernatant was removed and dimethyl sulfoxide (DMSO; Fisher BioReagents™, UK) was added to dissolve the formed formazan crystals in wells. At a wavelength of 570 nm, the optical densities of the solutions were then determined by microplate reader.

### Biofilm eradication activity

The eradication capacity of BiF2_5K7K against preformed biofilm was examined as previously described with some modifications [22, 23]. In brief, suspensions of *S. epidermidis* with a final density of 10^6^ CFU/mL were added to 96-well microplates, then covered with 96-peg lids (Nunc™ Immuno TSP Lids, Denmark). Following 24 h of incubation at 37°C, the peg lids were washed with 0.85% NaCl to remove culture media and non-adherent cells. Afterwards, the biofilms adherent to peg lids were incubated at 37°C for 24 h with different concentrations of peptide derivatives. The peg lids were subsequently washed and placed on new plates containing PBS. The biofilms on the lid were removed using a water bath sonicator (Bandelin SONOREX™, Germany). Biofilms were subsequently diluted and then placed on TSA. Minimum biofilm eradication concentrations at 50% and 90% (MBEC_50_ and MBEC_90_) refer to the concentration of the peptide at which established biofilm was eradicated by at least 50% and 90%, respectively [19, 20].

### Serum stability

The stability of BiF2_5K7K in the presence of human serum was assessed by evaluating the MIC values, as previously described [24]. Human serum was subjected to heat inactivation at 56°C for 30 min. Bacterial suspensions were then adjusted to a concentration of 10^6^ CFU/mL in MHB supplemented with 25% and 50% human serum. These suspensions were mixed with two-fold serially diluted concentrations of BiF2_5K7K (ranging from 0.98 to 250 µg/mL) and incubated at 37°C for 24 h. MICs were subsequently determined as described above. The study protocol was ethically approved by the Thammasat University Ethics Committee (COA No. 066/2562). All volunteers provided written informed consent.

### In vivo toxicity assay

The toxicity of peptide BiF2_5K7K against human lung fibroblast MRC-5 cell lines (ATCC CCL-171) was investigated by MTT colorimetric assay [16]. Briefly, MRC-5 cells were cultured in Minimum Essential Medium [MEM; supplemented with 10% heat-inactivated FBS and 1% Penicillin-Streptomycin] in 5% CO_2_ at 37°C. Following an overnight incubation, MRC-5 cells were diluted to 10,000 cells/well and placed in 96-well microplates. Subsequently, they were exposed to BiF2_5K7K at 0.98 to 250 µg/mL. After incubation for 24 h, MTT solution (0.4 mg/mL) was added to each well and incubated for an additional 3 h under 5% CO_2_ at 37°C. The supernatant was then removed and 100 µL DMSO added, and absorbance was measured at 570 nm using a microplate reader.

### Circular dichroism spectroscopy analysis

The conformational change of BiF2_5K7K in a membrane mimicking environment was determined by measuring CD spectra of the peptide at wavelengths 190 to 250 nm with a scanning speed of 10 nm/min by a Jasco-815 spectropolarimeter (Jasco, Japan). As previously described [25], the CD spectra were obtained using a quartz cell with a 1 mm path length at a temperature of 25°C. At a final concentration of 0.2 mg/mL, BiF2_5K7K was dissolved in three distinct membrane-mimicking environments, 10 mM PBS, 30 mM sodium dodecyl sulfate (SDS; Sigma-Aldrich, Japan), and 50% (v/v) 2,2,2-trifluoroethanol (TFE; Merck, USA). PBS served as the aqueous surrounding, while the SDS micelles mimic the negatively charged environment of bacterial membranes featuring an external surface with negative charges and an internal hydrophobic environment resembling phospholipid chains. TFE was utilized to simulate the hydrophobic compartment of microbial membranes as the fluorinated environment exhibits limited interaction with the molecule and induce intra-molecular interactions that frequently result in induced structuring, such as the formation of an alpha-helix structure [26]. An average of three scans were performed for each condition.

### Time-killing analysis

The time-dependent bactericidal activity of BiF2_5K7K against planktonic *S. epidermidis* was determined as previously described [23]. In brief, mid-log phase bacteria in MHB were exposed to BiF2_5K7K at MIC and/or MBC for 24 h at 37°C. The bacterial suspensions were obtained at specific time point (0, 2, 4, 6, 8 and 24 h), and 10-fold serially diluted with PBS and then plated on TSA. Untreated bacterial suspension was served as a control. After incubation, the number of CFUs was determined by colony count.

### Confocal laser scanning microscopy

An Amsterdam active attachment (AAA) model was used to assess the effectiveness of BiF2_5K7K to inhibit biofilm formation as previously described [22]. In a 24-well microplate, various concentrations of BiF2_5K7K were incubated overnight with 10^7^ CFU/mL of *S. epidermidis*. The plate was then covered with a sanitary stainless-steel lid containing double-glass coverslips. Following 24 h of incubation at 37°C, the biofilms were washed, and then 50 mM green Alexa-ConA (Sigma-Aldrich, USA) was applied for 30 min to stain the biofilms. After staining, biofilms were fixed for 3 h with 2.5% glutaraldehyde. Biofilm images were acquired using a confocal laser scanning microscope (CLSM; Carl-Zeiss/LSM800, Zeiss, Germany). The fluorescence intensity of each test was analysed using ZEN 2.1 (blue edition) software.

The effect of BiF2_5K7K against mature biofilm was also investigated. In short, overnight cultures of *S. epidermidis* were diluted to 10^7^ CFU/mL with TSB and further incubated at 37°C for 24 h to form biofilms. Next, wells were rinsed with sterile water and mature biofilms was treated with BiF2_5K7K at concentration of MBEC_50_ and further incubated for 24 h. Subsequently, biofilms were washed again, then stained with green Alexa-ConA and LIVE/DEADTM BacLight™ Bacterial Viability Kit (Invitrogen™, Thermo Fisher, USA) for 15 min. After staining, 2.5% glutaraldehyde was used to fixed biofilms, then observed under CLSM as described above.

### Transcriptional analysis

The expression level of *S. epidermidis* biofilm formation gene was investigated using a real-time reverse transcription-polymerase chain reaction [27, 28]. In brief, total RNA of *S. epidermidis* biofilm, both untreated and treated with BiF2_5K7K (at MBIC_50_ for 24 h), was extracted by a RNeasy PowerBiofilm Kit (Qiagen, USA) and immediately reverse transcribed to cDNA by iScript reserve transcription supermix (Biolabs, UK). RNAprotect Bacteria Reagent (Qiagen, USA) was used to preserve RNA during isolation. Remaining DNA was removed by treatment with DnaseI (Biolabs, UK). Real-time RT-PCR was done in duplicate using iTaq Universal SYBR Green Supermix (Bio-Rad, USA) with 5 ng of cDNA and 10 mM of forward and reverse primers [29, 30] (Table S1) using a CFX96 Real-Time PCR Detection System (Bio-Rad, USA). The cycling condition was started with an initial denaturation at 95°C for 2 min, followed by 50 amplification cycles consisting of 95°C for 30 s, 56°C for 1 min, and 72°C for 1 min. Subsequently, a melting curve was generated from 65°C to 95°C at intervals of 1 s at the end of each run. To analyse the relative quantitation of gene expression, 2^− ΔΔCt^ method was used, and 16S ribosomal subunit (rRNA) was served as the reference or housekeeping gene.

### Inhibitory activity of BiF2_5K7K on segments of silicone catheters

**Fig. 1:**
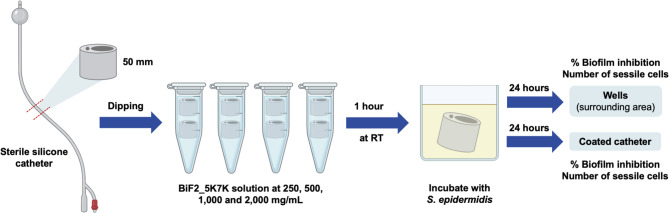
Workflow of peptide coating on catheters

The capacity of BiF2_5K7K to prevent *S. epidermidis* biofilm formation on a silicone catheter was determined using CV staining and colony count as previously described [31, 32] with some modifications (Fig. 1). CV staining was used to evaluate the development of biofilm on BiF2_5K7K-coated catheters and the surrounding area, while the viability of bacteria and the number of planktonic cells adherent to the catheter was assessed by colony count. Briefly, a sterile silicone catheter (Norta^®^, Malaysia) was sectioned into 50 mm segments which were then coated with BiF2_5K7K at concentrations of 250, 500, 1,000 or 2,000 mg/mL for 1 h by submerging the catheter segments, followed by overnight air-drying. The pre-coated catheters were subsequently placed in a 24-well microplate containing *S. epidermidis* suspension ($\sim$10^7^ CFU/mL in TSB) and incubated at 37°C. Following 24 h of biofilm formation, supernatant was collected for the colony count assay, and the wells were rinsed thrice with 0.85% NaCl.

To quantitate biofilm prevention, catheter segments were stained for 15 min with a 0.1% CV solution, then rinsed with 0.85% NaCl and resolved in 95% ethanol. Biofilm formation on catheter segments was measured by a microplate reader at absorbance of 570 nm. Wells were also stained with the CV solution, as previously described.

To evaluate the viability of sessile and planktonic *S. epidermidis* bacterial cells were removed from catheter segments by vortexing for 10 s, sonicating for 10 min, and vortexing again for 10 s. Cell suspensions were diluted and subsequently plated on TSA. The planktonic cells in wells were evaluated by ten-fold serially diluting collected supernatants and then plated on agar. After incubations, the CFUs were counted.

### Statistical analysis

The experiment was performed at least three times. The results were displayed as the mean ± standard deviation (SD). One-way ANOVA or Student’s *t*-test were used to analyse the data via GraphPad Prism (version 9.0). *p-value < 0.05, **p-value < 0.01, ***p-value < 0.001, and ****p-value < 0.0001 were considered significant.

## Results

### Novel antibiofilm peptides were designed and characterized

**Fig. 2:**
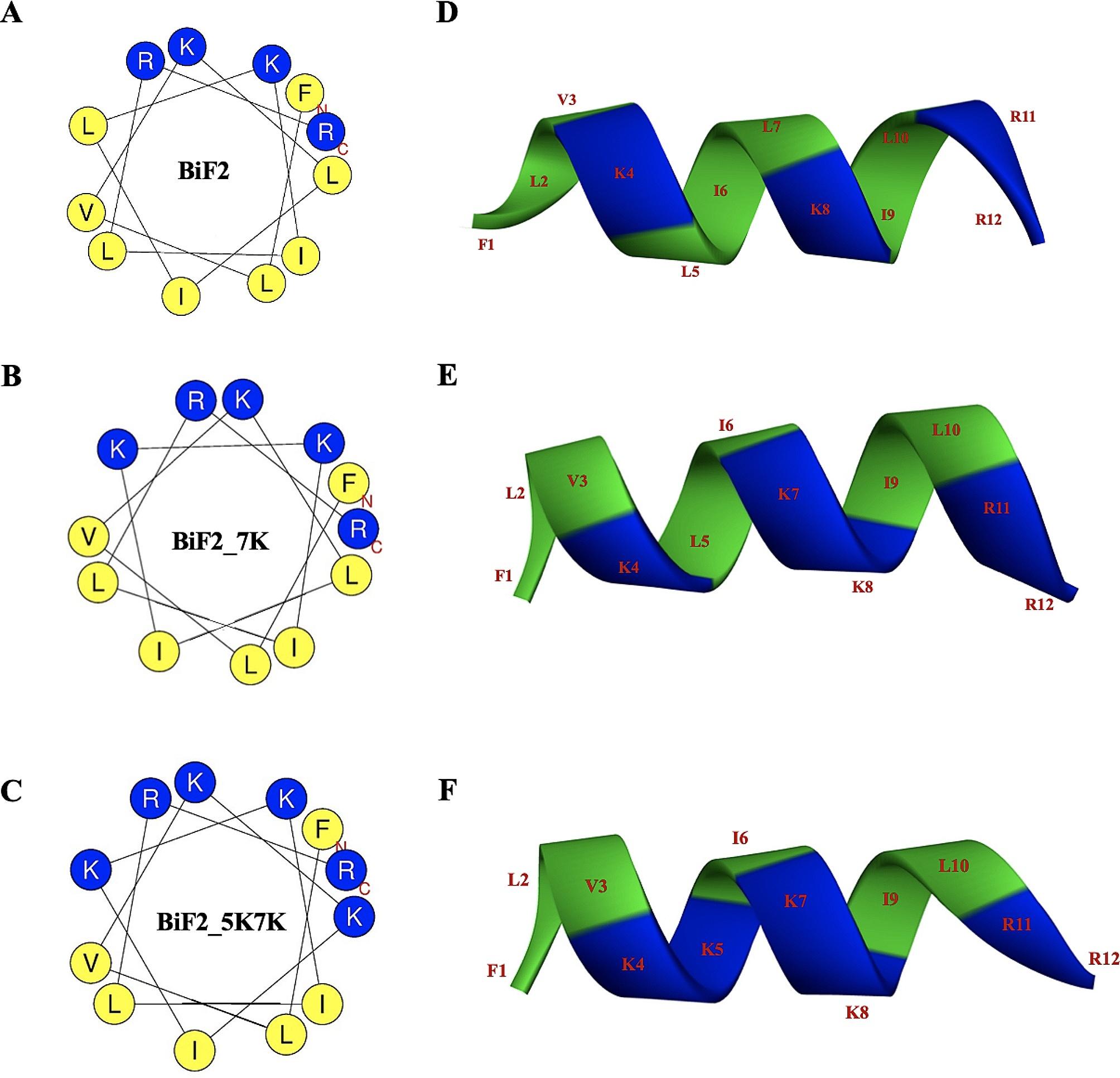
The helical wheel projections of peptides **A** BiF2, **B** BiF2_7K and **C** BiF2_5K7K. Positively charged and hydrophobic residues are shown in blue and yellow, respectively. The 3D structures are shown of **D** BiF2, **E** BiF2_7K and **F** BiF2_5K7K. Blue and green colors highlight positively charged and hydrophobic residues, respectively. The numbers represent the position of amino acid residues

Through a template-based design strategy, a 7-amino acid cationic BiF peptide was designed from the conserved, 18 α-helical sequence of antibiofilm peptides derived from diverse species (including frogs, bovines, sheep, fish, chickens, snakes, insects, and humans (Fig. S1). Hydrophobicity, amphipathicity and net charge were reported as the key parameters to designing novel peptides with enhanced activity while reducing toxicity [14]. Several studies have highlighted the significant influence of amphipathicity, often referred as peptide helicity, on antibiofilm activity, as it directly affects the mode of action [33]. Meanwhile, the antimicrobial activity of peptides is predominantly impacted by their charge and hydrophobicity [25]. As shown in Table 1, *in silico* analysis of the parent peptide, BiF, revealed a positive charge of +1 and high percentage (86%) of hydrophobic residues. To increase the overall positive charge and the associated antibiofilm activity, BiF was subsequently modified by hybridization at C-terminal segment with lipid binding motif, KILRR, from NaD1 peptide [17]. The newly obtained peptide, BiF2, with the net charge of +4 and 67% hydrophobicity, was predicted to be an α-helical amphipathic structure with perfect helical wheel projection in which the hydrophobic residue was located on one side and the charged residue on the other (Fig. 2).

Subsequently, the hydrophobic leucine residue on the polar face at positions 5 and 7 of BiF2 were substituted with lysine (L5K, L7K) to obtain two hybrid derivatives, BiF2_5K and BiF2_5K7K. These derivatives retained amphipathic α-helical structures with increased overall positive charge to +5 and +6, and reduced hydrophobic residues to 58% and 50%, respectively (Table 1; Fig. 2). These peptide derivatives were chemically synthesized, and their molecular weights (MWs) were confirmed by electrospray ionization-mass spectrometry (ESI-MS). The correlation between the calculated and measured MW for each peptide indicated accurate synthesis of the peptides [25].

**Table 1:** The amino acid sequences of the peptide derivatives and their corresponding physicochemical characteristics

Peptide	Sequence	aa^a^	Theoretical MW^b^	Measured MW^c^	Net charge	<H> ^d^	<µH>^e^	Pho%^f^
BiF	FLVKLIL	7	845.14	844.58	+1	-	-	86%
BiF2	FLVKLILKILRR	12	1512.00	1511.04	+4	0.784	0.603	67%
BiF2_7K	FLVKLI*K*KILRR	12	1527.01	1526.05	+5	0.560	0.716	58%
BiF2_5K7K	FLVK*K*I*K*KILRR	12	1542.02	1541.07	+6	0.336	0.679	50%

^a^ aa, number of amino acids

^b^ Theoretical MW, molecular weight calculated by https://aps.unmc.edu/prediction

^c^ Measured MW, molecular weight (g/mol) determined by mass spectroscopy (MS)

^d^ <H>, hydrophobicity and

^e^ <µH>, hydrophobic moment obtained from the website: http://heliquest.ipmc.cnrs.fr/

^f^ Pho%, the percentage of hydrophobic residues

-, No results were obtained

### BiF and its derivatives inhibited formation of *S. epidermidis* biofilm

**Fig. 3:**
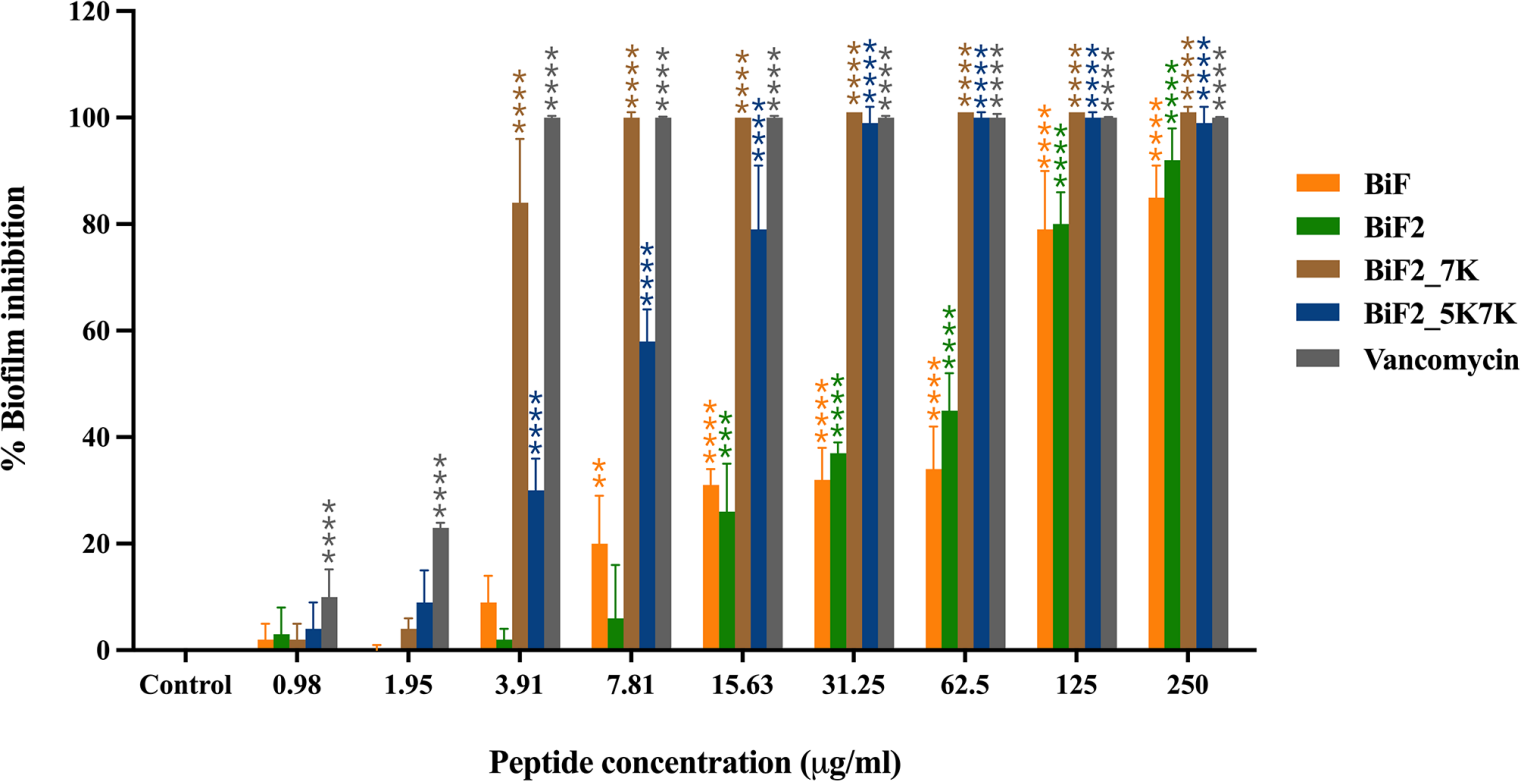
Inhibition of peptide derivatives against *S. epidermidis* biofilm compared to that of vancomycin. Data are presented as mean ± SD of at least three independent experiments. Significance was determined by one-way ANOVA (**p-value < 0.01, ****p-value < 0.0001)

According to the result of inherent biofilm formation capacity by CV staining assay, *S. epidermidis* ATCC 35984 was classified as a strong biofilm-producing strain, as evidenced by its OD570 being four times higher than the ODc (data not shown). The ability of BiF and its derivatives to prevent biofilm formed by *S. epidermidis* was further investigated. The concentrations of peptide which inhibited 50% and 90% of biofilm formation were defined as MBIC_50_ and MBIC_90_, respectively. As shown in Fig. 3, all peptide derivatives displayed a dose-dependent inhibition of biofilm development. The parent peptide, BiF, exhibited low biofilm inhibitory activity. However, the lipid binding motif-conjugated peptide (BiF2) effectively prevented the formation of biofilm with MBIC_50_ and MBIC_90_ values of 125 and 250 µg/mL, respectively (Table 2). The modified peptides, BiF2_7K and BiF2_5K7K, exhibited higher biofilm inhibitory activity with MBIC_50_ values of 3.91 and 7.81 µg/mL, respectively, and MBIC_90_ values of 7.81 and 31.25 µg/mL, respectively. Vancomycin, a glycopeptide antibiotic known for its inhibitory activity against *S. epidermidis* biofilms, served as a positive control to assess biofilm inhibition [34]. The MBIC_50_ and MBIC_90_ of vancomycin were both 3.91 µg/mL.

**Table 2:** The activities of peptide derivatives against *S. epidermidis* biofilm

Peptide	MBIC_50_^a^ (µg/mL)	MBIC_90_^b^ (µg/mL)
BiF	125	>250
BiF2	125	250
BiF2_7K	3.91	7.81
BiF2_5K7K	7.81	31.25

^a^ MBIC_50_ and ^b^ MBIC_90_, are defined as the minimum concentration of peptide that can inhibit biofilm formation more than 50% and 90%, respectively

### Modified peptide derivatives showed improved efficacy and safety

**Fig. 4:**
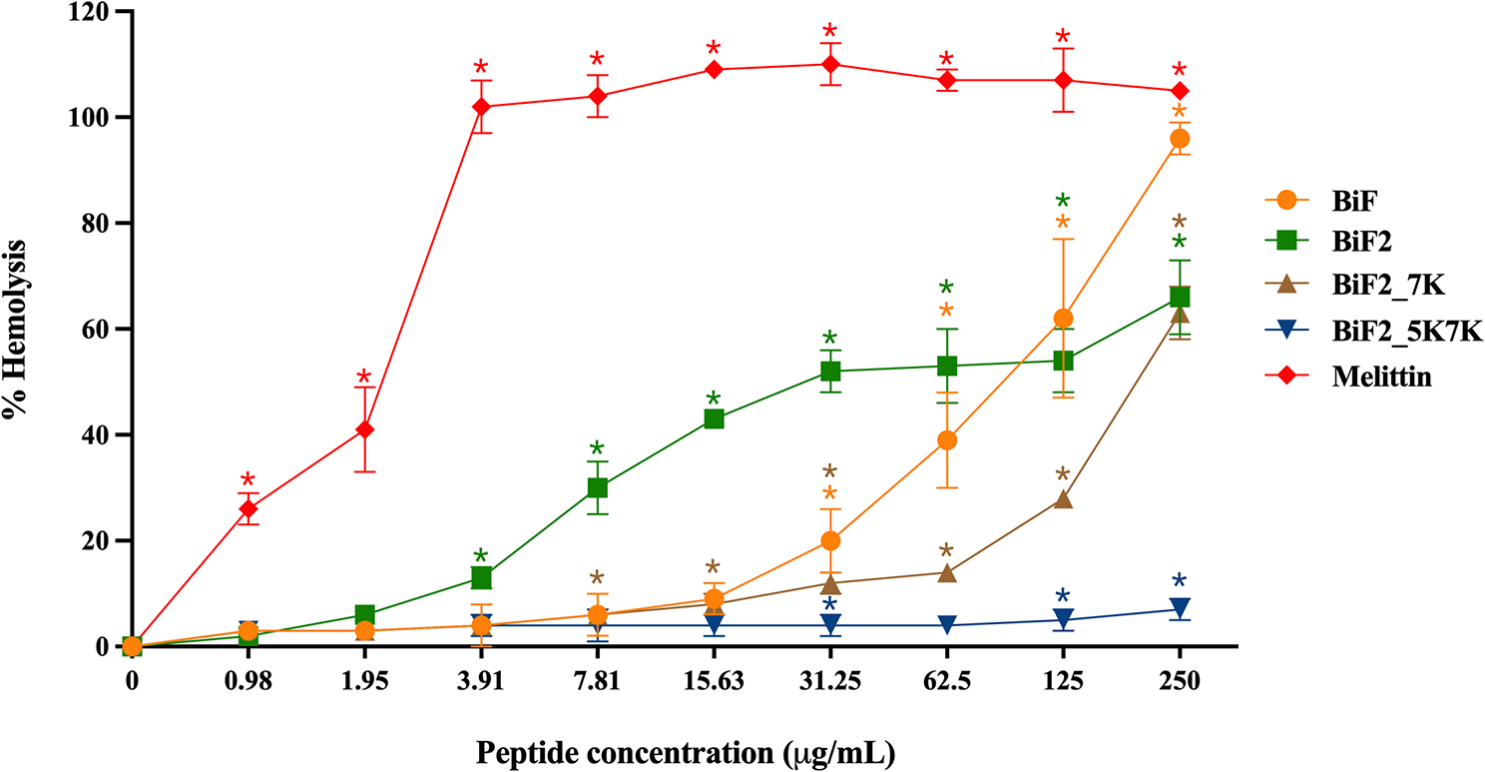
The hemolytic activity of peptide derivatives on hRBCs. Tests were done in triplicate and reported as mean ± SD. Statistical significance was evaluated using one-way ANOVA (*p-value < 0.05)

The antibacterial activity of BiF and its derivatives against planktonic biofilm-forming bacteria were determined. When tested against target bacteria, the parent peptide BiF showed no antibacterial activity, while the modified peptides (with one and two lysine substitutions, BiF2_7K and BiF2_5K7K) had broad-spectrum antibacterial activity against biofilm-forming bacteria at concentrations ranging from 7.81 to 250 µg/mL (Table 3). Moreover, these modified peptides also demonstrated lower hemolytic activity than did the parent peptide as shown in Fig. 4. At a concentration of 31.25 µg/mL, more than 20% of hRBCs were lysed by BiF, and the lysis reached 100% at a concentration of 250 µg/mL. In contrast, BiF2_7K at the maximum concentration tested (250 µg/mL) displayed 60% hemolytic activity. Remarkably, BiF2_5K7K displayed minimal hemolytic effect, measuring less than 10%, even at its maximum concentration. In contrast, the membrane-lytic melittin peptide, derived from bee venom, used in hemolytic and antibacterial experiments as a positive control, exhibited potent antibacterial activity within the range of 3.91 to 15.63 µg/mL. Furthermore, at 3.91 µg/mL, melittin completely lysed hRBCs.

The therapeutic index (TI) is widely used for evaluating a peptide’s efficacy and safety [35]. It is the ratio between the minimum hemolytic concentration (MHC, the concentration causing 10% hemolysis) and the geometric mean (GM) value of the MIC [15]. Greater TI values indicate that the peptide is more selective and specific for bacterial-like membranes than those of mammals [25]. As shown in Table 3, BiF and BiF2, as well as the positive control melittin, exhibited very narrow therapeutic windows (TI less than 0.2). While, both lysine-substituted peptides (BiF2_7K and BiF2_5K7K) had higher TIs against all tested biofilm-forming bacteria than did the two parent peptides, and the TI of BiF2_5K7K was higher than that of BiF2_7K.

Based on its high inhibitory levels of biofilm-formation and low hemolytic activity, BiF2_5K7K was shown to be a promising candidate peptide against biofilm-forming bacteria. This assessment includes biofilm-forming *S. epidermidis*, widely known as one of the most prevalent causes of biofilm-related, medical device infections [3]. At subinhibitory concentrations (<15.63 µg/mL), BiF2_5K7K was still able to prevent the formation of biofilm by *S. epidermidis*.

**Table 3:** The antibacterial activities (MIC and MBC values) of peptide derivatives against both gram-positive and gram-negative bacteria

Bacterial strains		MIC (MBC) (µg/mL)				
	**Strain**	**BiF**	**BiF2**	**BiF2_7K**	**BiF2_5K7K**	**Melittin**
***Gram-positive bacteria***						
*Staphylococcus epidermidis*	ATCC 35984^a^	>250 (>250)	31.25 (62.5)	7.81 (7.81)	15.63 (31.25)	15.63 (15.63)
*Staphylococcus aureus*	ATCC 25923^a^	>250 (>250)	62.5 (62.5)	15.63 (15.63)	>250 (>250)	3.91 (3.91)
*Bacillus cereus*	ATCC 11778^a^	>250 (>250)	15.63 (15.63)	7.81 (7.81)	250 (250)	3.91 (3.91)
*Listeria monocytogenes*	10403s	>250 (>250)	15.63 (31.25)	7.81 (7.81)	31.25 (62.5)	3.91 (3.91)
***Gram-negative bacteria***						
*Pseudomonas aeruginosa*	ATCC 27853^a^	>250 (>250)	15.63 (31.25)	15.63 (15.63)	62.5 (125)	15.63 (15.63)
*Salmonella* Typhimurium	ATCC 13311^a^	>250 (>250)	62.5 (62.5)	7.81 (15.63)	31.25 (62.5)	3.91 (3.91)
*Escherichia coli*	ATCC 25922^a^	>250 (>250)	31.25 (31.25)	31.25 (31.25)	250 (250)	31.25 (31.25)
*Acinetobacter baumanii*	MT strain^b^	>250 (>250)	62.5 (62.5)	7.81 (7.81)	31.25 (31.25)	7.81 (7.81)
MHC^c^ (µg/mL)		15.63	1.95	15.63	>250	<0.98
GM +^d^		>250	31.25	9.77	199.22	6.84
TI +^e^		0.03	0.06	1.60	2.51	0.07
GM-^f^		>250	42.97	15.63	93.75	14.65
TI-^g^		0.03	0.05	1	5.33	0.03

^a^ ATCC, the American Type Culture Collection

^b^ MT strain, bacterial strain provided from Department of Medical Technology, Faculty of Allied Health Sciences, Thammasat University

^c^ MHC, minimum hemolytic concentration, indicated the peptide concentration causing 10% hemolysis of hRBCs. When no 10% hemolysis was detected at 250 µg/mL, a value of 500 µg/mL was used to calculate the therapeutic index. When no 10% hemolysis was observed at a concentration of 0.98 µg/mL, a value of 0.49 µg/mL was utilized for calculating the therapeutic index

^d^ GM + and ^f^ GM-, the geometric mean of MIC values from all gram-positive and -negative bacterial strains, respectively

^e^ TI + and ^g^ TI-, therapeutic index is the ratio of the MHC to the geometric mean of MICs from all gram-positive and -negative bacterial strains, respectively

### BiF2_5K7K showed no toxicity to lung fibroblasts

**Fig. 5:**
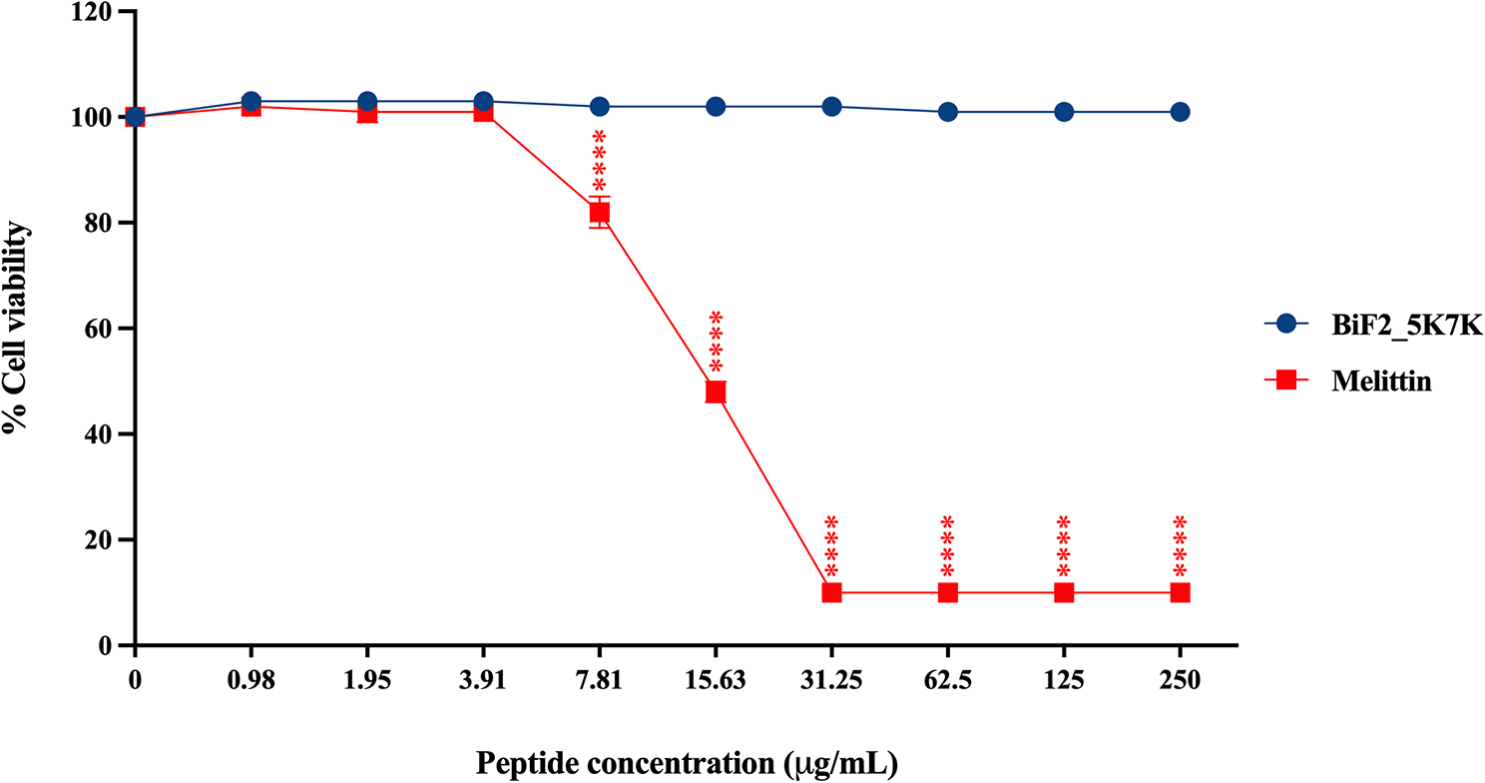
The cytotoxicity of BiF2_5K7K and melittin toward MRC-5 human embryonal lung fibroblast cell line. Experiments were performed in triplicate and data are reported as mean ± SD. *p-value < 0.05, **p-value < 0.01, ***p-value < 0.001, and ****p-value < 0.0001 indicate significant differences

Since BiF2_5K7K demonstrated low hemolytic effect against hRBCs, its toxicity on the MRC-5 human embryonal lung fibroblast cell line was assessed through a MTT viability assay. In Fig. 5, BiF2_5K7K was not toxic (cell viability > 99%) toward the MRC-5 cell line even at the maximum concentration tested (250 µg/mL). In contrast, melittin, the positive control peptide, exhibited a strong dose-dependent cytotoxic effect.

### BiF2_5K7K exhibited time- and concentration-dependent antibacterial activity and maintained its efficacy in human serum

**Fig. 6:**
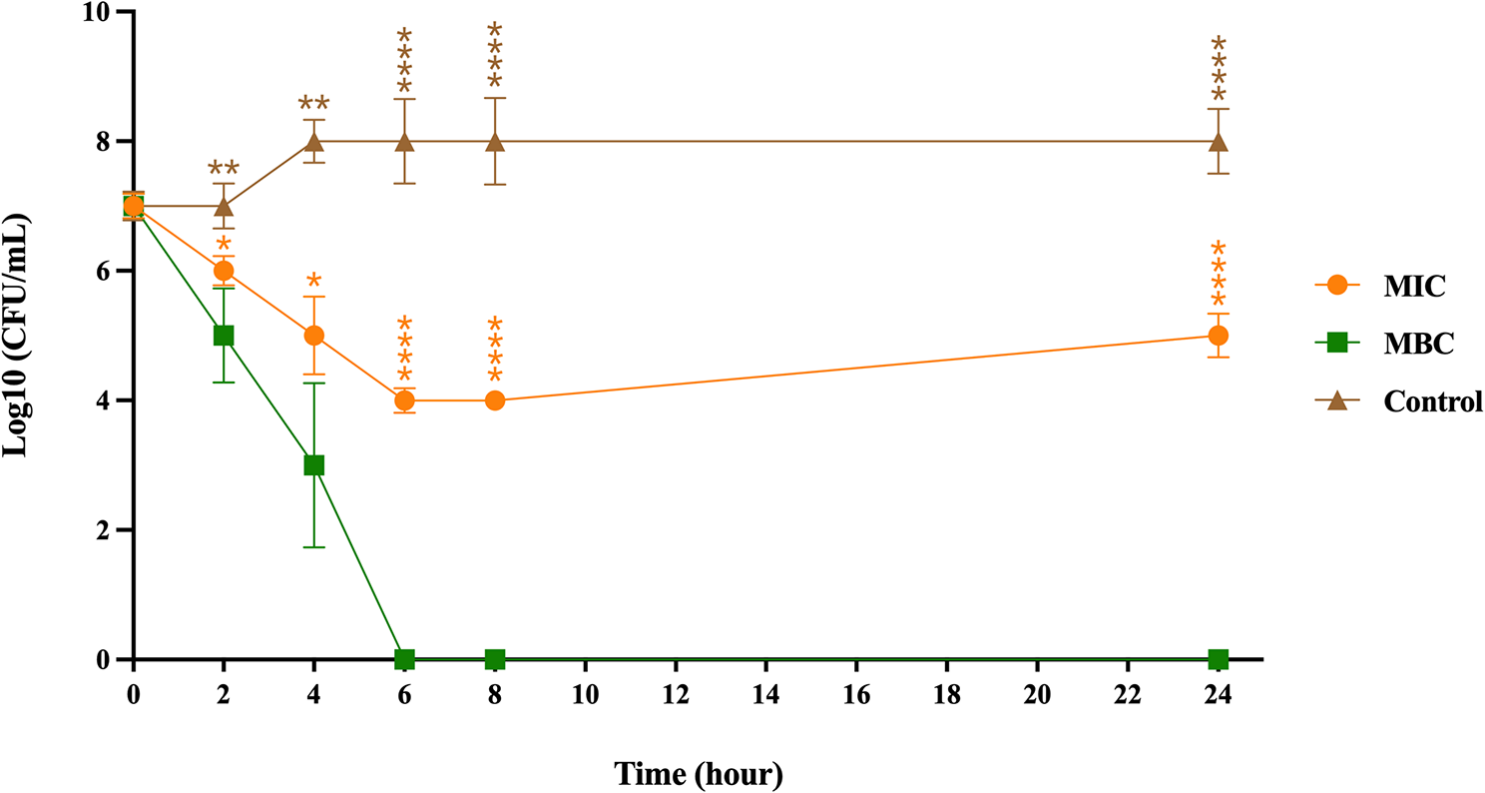
The time-dependent bactericidal activity of BiF2_5K7K at both MIC and MBC against *S. epidermidis* was evaluated at 0, 2, 4, 6, 8, and 24 h. Experiments were conducted in triplicate, and results are presented as the mean ± SD. Statistical significance was denoted by *p-value < 0.05, **p-value < 0.01, ***p-value < 0.001, and ****p-value < 0.0001, indicating significant differences in the data

The antibacterial activity of BiF2_5K7K against *S. epidermidis* was carried out with BiF2_5K7K at both MIC and MBC concentrations (Fig. 6). In comparison to the untreated control, both MIC and MBC concentrations of BiF2_5K7K significantly decreased the number of *S. epidermidis* (reaching approximately 10^3^ CFU/mL) within 4 h. At MBC, BiF2_5K7K completely killed *S. epidermidis* (more than 99% killing) within 6 h (while regrowth was observed at MIC after 8 h). In the presence of 25% and 50% human serum, BiF2_5K7K exhibited no change of antibacterial activity against *S. epidermidis* (MIC = 15.63 µg/mL), suggesting that BiF2_5K7K exhibits tolerable stability when exposed to human serum.

### BiF2_5K7K displayed α-helical structure in membrane-mimicking environment

**Fig. 7:**
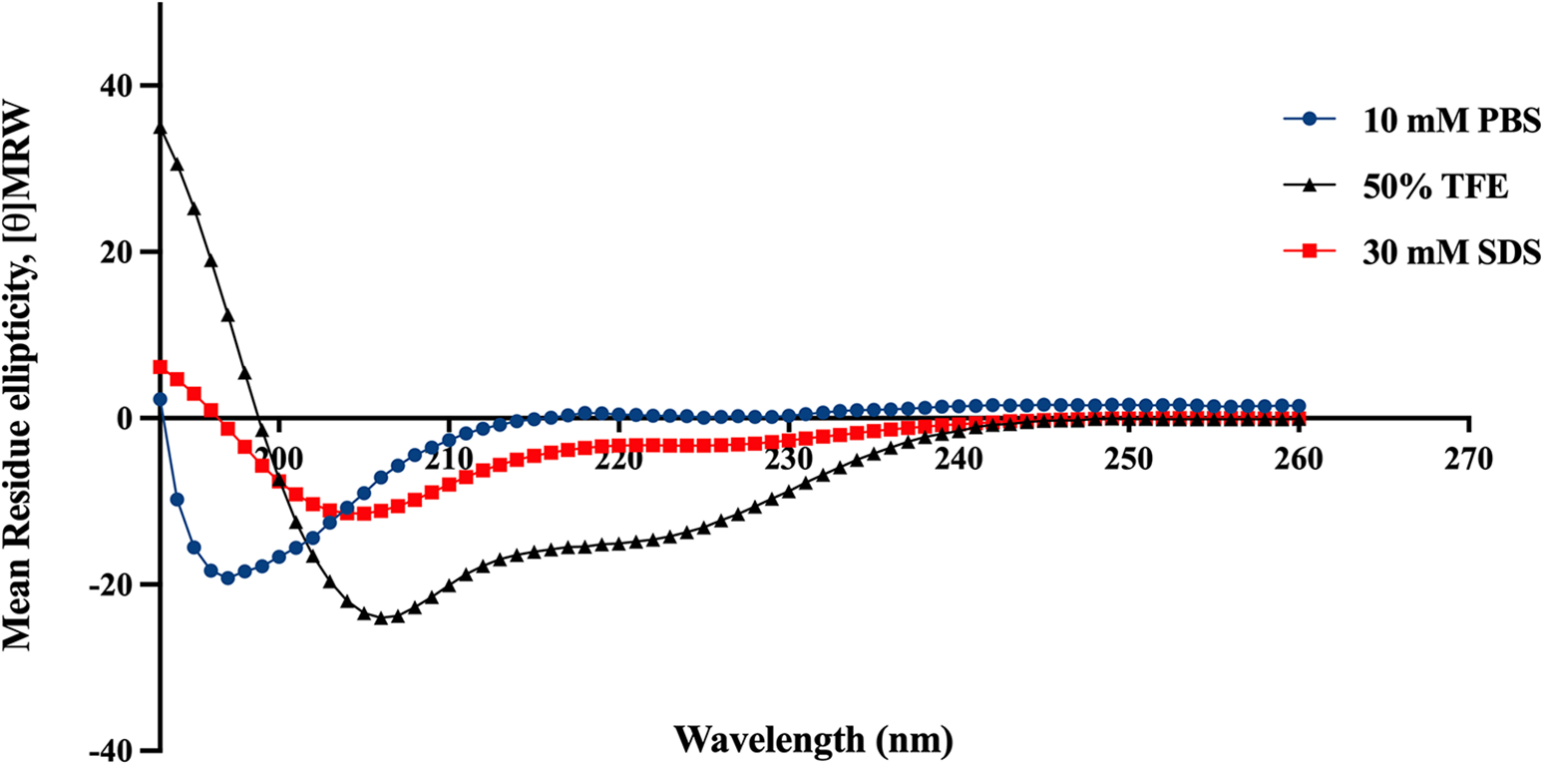
Circular dichroism spectra of BiF2_5K7K in 10 mM PBS (blue), 50% TFE solution (black) and 30 mM SDS (red)

The secondary structure of BiF2_5K7K was examined by CD spectroscopy in various environments simulating the bacterial membranes (Fig. 7). In 30 mM SDS and 50% TFE solutions, BiF2_5K7K adopts a stable amphipathic α-helical structure as shown by negative peaks at approximately 208 and 222 nm. In an aqueous environment (10 mM PBS), the spectra of the peptide revealed a negative peak near 200 nm, indicating an unordered or random coil structure.

### BiF2_5K7K inhibited *S. epidermidis* biofilm formation by killing activity and altering biofilm gene expression

**Fig. 8:**
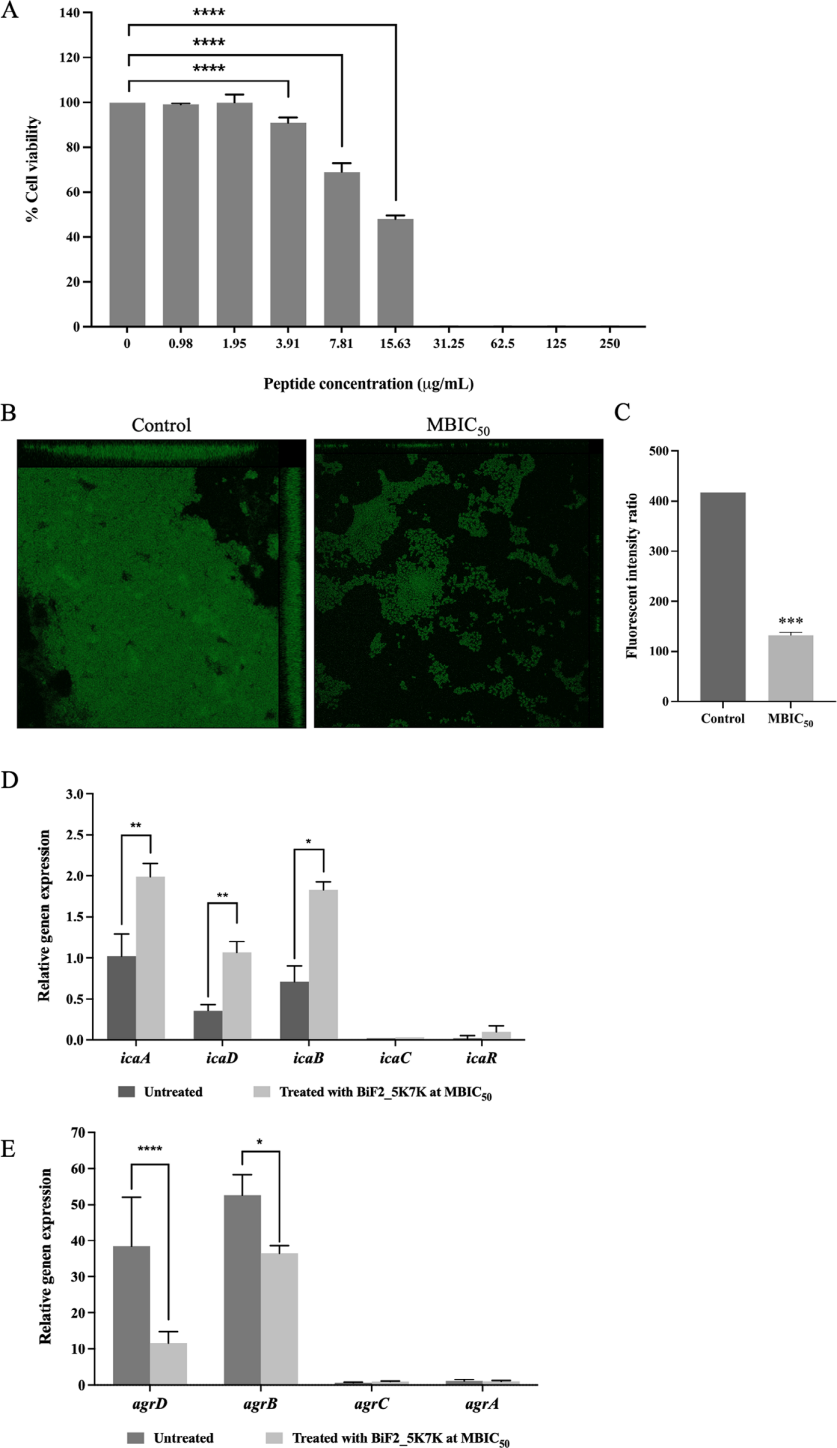
Biofilm-formation inhibitory activity of BiF2_5K7K on *S. epidermidis* biofilm. **A** Biofilm cell viability after treatment with 0.98–250 µg/mL of BiF2_5K7K. **B** Confocal imaging of *S. epidermidis* biofilm treated with BiF2_5K7K at MBIC_50_. **C** The fluorescence intensity ratio of Alexa Flour^®^ 488 stained biofilms. **D** and **E** The relative gene expression of *S. epidermidis* polysaccharide production and quorum-sensing genes, respectively. The data presented represent the mean ± SD of at least three independent experiments. Statistical significance is denoted by *p-value < 0.05, **p-value < 0.01, ***p-value < 0.001, and ****p-value < 0.0001, indicating the presence of significant differences in the results

The viability of *S. epidermidis* cells embedded in a biofilm and exposed to various concentrations of BiF2_5K7K demonstrated a concentration-dependent bactericidal activity, as shown in Fig. 8A. It was observed that at a concentration that inhibits bacterial growth, or MIC (15.63 µg/mL), *S. epidermidis* biofilm cells remained viable. This result indicated a significant difference in the response between planktonic and biofilm cells to the peptide treatment. At higher concentrations (>15.63 µg/mL), there was no growth of biofilm cells, suggesting that the peptide had a bactericidal effect operating through a mechanism similar to its action on planktonic cells. These results aligned with the biofilm inhibitory activity shown by the crystal violet staining (Fig. 3), that at higher concentrations, the peptide exerted a killing effect which prevented biofilm formation. However, at subinhibitory concentrations, crystal violet staining showed that the biofilm inhibitory activity of BiF2_5K7K allowed the biofilm cells to remain viable.

The impact of BiF2_5K7K at a subinhibitory concentration [MBIC_50_ (7.81 µg/mL)] on biofilm formation was further evaluated by visualizing biofilm matrix labelled with Alexa Fluor^®^ 488. As shown in Fig. 8B, the reduction in *S. epidermidis* biofilm after treatment with 7.81 µg/mL BiF2_5K7K was evident through the decrease in the green fluorescence emitted by Alexa Fluor^®^ 488, comparable to that of the untreated sample. These findings were further validated by assessing the fluorescence intensity (Fig. 8C). For the *S. epidermidis* biofilm treated with BiF2_5K7K at MBIC_50_, this was found to be significantly lower than that of the untreated control (*p*-value < 0.001). The overall results indicated that BiF2_5K7K at MBIC_50_ did not inhibit bacterial growth but did reduce biofilm development by *S. epidermidis*.

The effect of BiF2_5K7K on polysaccharide intercellular adhesin (PIA)-producing genes (*icaA*, *icaD*, *icaB*, *icaC*, and *icaR*) and *agr* quorum-sensing genes (*agrD*, *agrB*, *agrC*, and *agrA*) is shown in Fig. 8D and E, respectively. Treatment with BiK2_5K7K at MBIC_50_ significantly upregulated *icaA*, *icaD*, and *icaB* genes expression, with the relative expression level greater than 1 at 24 h (Fig. 8D); no alteration was noticed in levels of *icaC* or *icaR* genes. In contrast, expression of the quorum-sensing genes *agrD* and *agrB* was reduced after 24 h of BiF2_5K7K treatment of *S. epidermidis* biofilm compared to that of the untreated control (Fig. 8E). However, neither *agrC* nor *agrA* gene expression changed at all over this time period.

### BiF2_5K7K destroyed preformed *S. epidermidis* biofilm

**Fig. 9:**
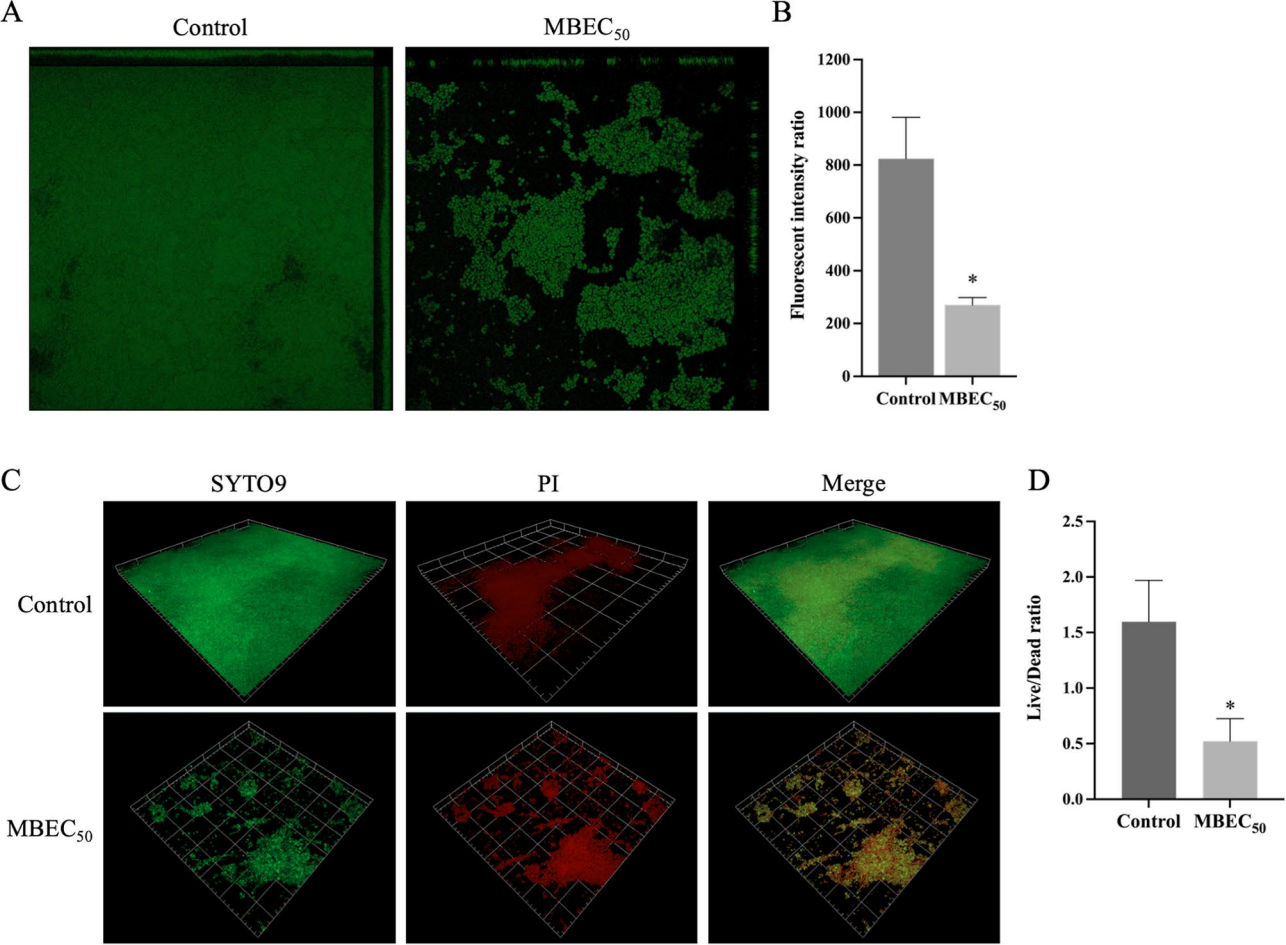
Biofilm eradication activity of BiF2_5K7K on *S. epidermidis* biofilm. **A** Confocal imaging of preformed *S. epidermidis* biofilms after treatment with BiF2_5K7K at MBEC_50_. **B** The fluorescence intensity ratio of Alexa Flour^®^ 488 stained biofilms. **C** Live/dead results of *S. epidermidis* biofilms after treatment with BiF2_5K7K at MBEC_50_. Live cells stained with SYTO 9 (green) while dead cells stained with propidium iodide (red). **D** The fluorescence intensity ratios of live/dead cells analysed by ZEN 2.1. The presented data represents the mean ± SD derived from a at least three independent experiments. Statistical significance is indicated by *p-value < 0.05, **p-value < 0.01, ***p-value < 0.001, and ****p-value < 0.0001, highlighting the presence of notable differences in the findings

BiF2_5K7K revealed a concentration-dependent eradication activity on established biofilm of *S. epidermidis* (Fig. S2). More than 90% of mature biofilm was eradicated after 24 h of peptide treatment at a dosage of 125 µg/mL (MBEC_90_), while the MBEC_50_ was 31.25 µg/mL. The destruction capability of BiF2_5K7K on mature biofilm was confirmed by confocal imaging analysis. After treatment with BiF2_5K7K at MBEC_50_, a significant decrease in the *S. epidermidis* biofilm matrix was revealed by its lower fluorescence intensity (Fig. 9A and B). In parallel, *S. epidermidis* biofilm cells viability after exposure to peptide was determined by live/dead staining. Established *S. epidermidis* biofilm treated with BiF2_5K7K at MBEC_50_ exhibited a reduction in green fluorescence intensity, with the majority of bacteria staining red and their distribution uneven as compared to control (Fig. 9C). The fluorescence intensity ratio (live to dead cells) demonstrated that there was a significant difference between mature biofilm treated with BiF2_5K7K and untreated (*p*-value < 0.01) (Fig. 9D). Based on these findings, it appeared that BiF2_5K7K was capable of damaging mature *S. epidermidis* biofilm.

### BiF2_5K7K prevented biofilm formation on silicone catheter

**Fig. 10:**
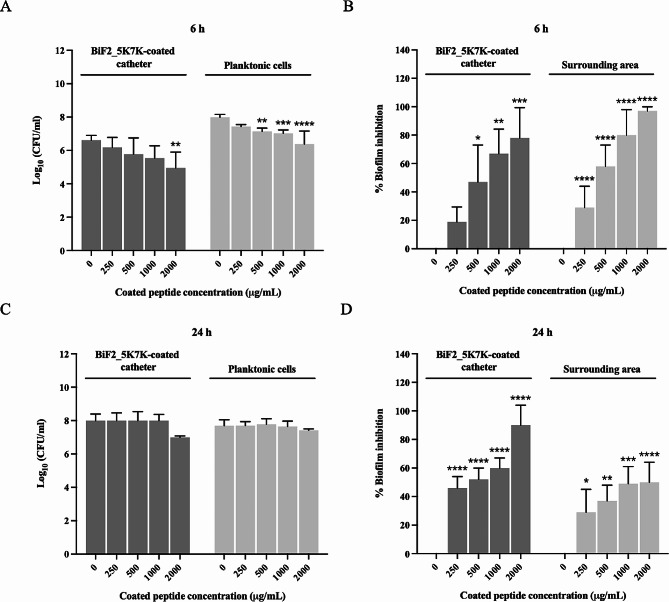
Antibiofilm activity of BiF2_5K7K on silicone catheter. **A** and **C** Bactericidal effect of BiF2_5K7K-coated catheters against sessile and planktonic *S. epidermidis* after 6 and 24 h of incubation, respectively. **B** and **D** Biofilm inhibitory activity on BiF2_5K7K-coated catheter and surrounding area, respectively. The experiments were done at least in triplicate; data are represented as the mean ± SD. *p-value < 0.05, **p-value < 0.01, ***p-value < 0.001, and ****p-value < 0.0001 indicate significant differences

The inhibitory activity of BiF2_5K7K against *S. epidermidis* biofilm formation on a silicone catheter was evaluated as a model for medical devices. A silicone catheter was coated with BiF2_5K7K by immersion in peptide solution at concentrations of 250, 500, 1,000 or 2,000 µg/mL, and the amount of released peptide from the catheter was measured by fluorescamine protein assay. There were approximately 1.5, 4, 14, and 32 µg/mL of peptide released, respectively (data not shown). Within 6 h, coated catheters with BiF2_5K7K were able to limit the adherence of *S. epidermidis* to the catheter surface, particularly those coated with BiF2_5K7K at 2,000 µg/mL (Fig. 10A). Coating resulted in a significant reduction in the number of sessile bacteria (from 10^7^ to 10^5^ CFU/mL). Peptide released from the coated catheters significantly inhibited the growth of planktonic cells (Fig. 10A). Interestingly, the inhibition of biofilm development on the BiF2_5K7K-coated catheter and surrounding area was also observed (Fig. 10B), indicating the capability of BiF2_5K7K not only directly inhibit biofilm formation on the catheter but also to diffuse from the coated catheter and inhibit biofilm formation within the surrounding environment (wells).

Nevertheless, after 24 h, the attachment of sessile bacteria to the catheter surface and the growth of planktonic bacteria could only be inhibited on catheters coated with 2,000 µg/mL of BiF2_5K7K (Fig. 10C). At all other concentrations, this effect was not observed. Even though the other BiF2_5K7K-coated catheters were unable to inhibit attachment and growth of bacteria, they substantially inhibited the development of biofilm both on the catheter itself and in the surrounding area (Fig. 10D). These findings were supportive of the capability of BiF2_5K7K to prevent *S. epidermidis* biofilm development on catheters. In this experiment, vancomycin was served as the antibiotic control (Fig. S3).

## Discussion

Medical devices play a crucial role in prolonging and enhancing quality of life, notably among the elderly [36]. During and after implantation, medical devices can become contaminated with normal flora like *S. epidermidis* as well as other pathogenic organisms, causing chronic infections, device failure and high mortality rates [5, 10]. The adherence of biofilm-forming microbes (such as *S. epidermidis*) to medical devices makes this problem even worse [37], since biofilms are surrounded by an EPS (composed predominantly of polysaccharide, extracellular DNA (eDNA) and protein) which serves as a physical barrier, protecting the bacteria within the biofilm from host defences and antimicrobial agents [10]. Consequently, the development of novel agents for treatment of biofilm-associated infections, especially medical device-related infections, is much needed.

Rationally designed peptides have become a promising strategy for both inhibiting formation of biofilms and/or eradicating established biofilms. This is due to their potent and rapid antibiofilm and antimicrobial activity against both planktonic and biofilm-embedded cells [10]. Additionally, they have a low likelihood of inducing bacterial resistance due to their distinct mechanisms of action compared to conventional antibiotics [11, 12]. Some antibiofilm peptides incorporated onto medical device surfaces have been shown to reduce initial attachment and, thus, biofilm development [10, 38, 39]. Moreover, antibiofilm peptides can be combined with antibiotics or other antibiofilm agents that act with different mechanisms and/or target-distinct biofilm features, and so improve overall antibiofilm activity [10, 16].

By the sequence alignment of α-helical peptides with antibiofilm activity, a consensus amphipathic sequence (FLVKLIL) rich in hydrophobic residues were retrieved and served as a core antibiofilm peptide, BiF, for further modification. Peptide hybridization provides an alternative method for promoting the activity and specificity of peptides while minimizing their toxicity. In recent years, reports of combinations of peptides and motifs with specific functions have increased [40]. The hybrid peptide R-FV-I16 was derived from RI16, an antimicrobial peptide, and combined with the antibiofilm motif FV7. This hybrid peptide has potent antimicrobial and antibiofilm activities against both gram-positive and -negative pathogenic bacteria, as well as reduced hemolytic activity and cytotoxicity [15]. We therefore introduced a highly positively charged lipid binding site motif (KILRR motif from the NaD1 peptide) into the sequence of BiF at the C-terminus to obtain a novel hybrid peptide (BiF2) with higher positive charge and lower hydrophobicity. A previous study demonstrated that NaD1, an antifungal plant defensin, binds specifically to phospholipids, particularly phosphatidylinositol 4,5-bisphosphate (PIP2), via the K4 residue and KILRR motif [17]. The incorporation of BiF with a lipid binding site motif not only enhanced antibiofilm activity but also antibacterial activity. This enhancement is likely due to increased interaction between the lipid binding site of the peptides and the lipids present in bacterial cell membranes [41]. Compared to the parent peptide, this incorporation did not lead to significant differences in toxicity against human red blood cells. This observation suggested that the decrease in hydrophobicity was not substantial enough to significantly enhance cell selectivity.

A study by Von Borowski and colleagues revealed that lysine (K) and phenylalanine (F) are the most found amino acids found in antibiofilm peptides [42]. Furthermore, their substitution with cationic amino acid residues, such as lysine or arginine (R), can increase the positive charge of these peptides and enhance antimicrobial activity [43, 44]. Subsequently, antibiofilm activity as one mechanism of action of these peptides was enhanced along with their antimicrobial activity. There is evidence that membrane disruption is the primary mechanism underlying their antibiofilm action [43]. Two hydrophobic amino acids at positions 5 and 7 of BiF2 were substituted with positively charged lysines (L5K, L7K) in order to increase the positive charge and induce an amphipathic structure of peptide characterized by a hydrophobic surface on one side and a hydrophilic surface consisting of positively charged amino acid residues on the other. Lysine was chosen over arginine because of its high helical propensity and lower tendency to cytotoxicity [44]. As a result, BiF2_7K and BiF2_5K7K were obtained and both showed remarkable antibiofilm and antibacterial activity. Strikingly, their effect toward human red blood cells was significantly decreased in comparison to the parent BiF2 peptide. These hybrid peptides and their derivatives exhibited broad-spectrum activity against both gram-positive and -negative bacteria. In contrast, the parent peptide (BiF) displayed negligible antibacterial activity. The novel designed peptide BiF2_5K7K, which integrates the parent sequence with a lipid binding site motif and substitutes the amino acid at positions 5 and 7 with lysine, demonstrated higher antibiofilm and antibacterial activities while mitigating toxicity when compared to that of parent peptide. This enhancement is attributed to the optimization of the hydrophobicity, amphipathicity, and net charge of the parent peptide.

The TI value serves as a parameter in assessing the efficacy and safety of these peptides for clinical applications [45]. Among the peptide derivatives, BiF2_5K7K exhibited the highest TI values against both gram-positive and -negative bacteria compared to others, even though BiF2_7K demonstrated greater antibiofilm and antibacterial activities. The TI value of peptides is influenced by their hemolytic effects, and thus helps ensure their safety towards mammalian cells and guide their development [16]. In order to evaluate its potential for further use, a study of the cytotoxicity of BiF2_5K7K against MCR-5 cells was conducted. The results indicated that BiF2_5K7K was not toxic towards mammalian cells. The stability of peptides in serum is crucial for evaluating their effectiveness in environments resembling in vivo conditions [46]. When exposed to 25% and 50% human serum, BiF2_5K7K maintained its antimicrobial activity, indicating its potential applicability in biological settings. The staphylocidal activity of BP2, a small synthetic amphipathic peptide designed based on the lipopolysaccharide-binding domains found in various naturally occurring proteins, showed slightly changes when exposed to human plasma [47].

BiF2_5K7K effectively inhibited *S. epidermidis* biofilm development by more than 50% at MBIC_50_ (7.81 µg/mL). This inhibition was further confirmed by CLSM images, which demonstrated a reduction of the biofilm matrix. Moreover, at higher concentrations, BiF2_5K7K exerted its biofilm-inhibiting effect by eradicating bacteria before biofilm formation, indicating the biofilm inhibition is achieved through its bactericidal activity. Therefore, to achieve complete inhibition (greater than 90% inhibition or MBIC_90_), a significantly higher concentration was necessary. This finding suggested that at subinhibitory concentration, BiF2_5K7K may have employed an alternative mechanism to combat biofilms rather than relying only on bactericidal actions [43].

It has been observed that there is no consistent correlation between antibacterial and antibiofilm activities. While certain peptides demonstrate antibiofilm effects without affecting planktonic bacteria [10, 13], others possess the capacities for both antibacterial activity and inhibition and/or elimination of biofilms [48]. BiF2_5K7K is a peptide exhibiting dual functionality, encompassing both antibacterial and antibiofilm activities. LL-37, a well-known peptide with antibacterial and antibiofilm activities, displays broad-spectrum antibacterial activity. Moreover, it has the capability to inhibit *P. aeruginosa* biofilm development through the promotion of twitching motilities, downregulation of genes essential responsible for biofilm formation, and the modulation of two key quorum-sensing systems [49]. The results of this study corroborated previous observations, indicating that the interference with genes responsible for *S. epidermidis* biofilm formation might serve as a central mechanism underlying the biofilm-inhibiting property of BiF2_5K7K.

The primary component within the *S. epidermidis* biofilm matrix is an extracellular polysaccharide referred to as polysaccharide intercellular adhesin (PIA) or poly-N-acetylglucosamine (PNAG), which is a product of the intracellular adhesion (*ica*) operon that composes *icaA*, *icaD*, *icaB*, *icaC*, and *icaR* (regulator of the *ica* locus) genes [50]. The *icaA* and *icaD* genes encode transmembrane protein N-acetyl-glucosamine oligomers. The longer oligomer chains are transported across the cytoplasmic membrane by the IcaC protein and deacetylated by the IcaB protein for cell surface attachment. The *icaR* gene has a regulatory role in repressing transcription at the *ica* locus [51, 52]. Following exposure to BiF2_5K7K, gene expression within the *ica* operon includes upregulation of the *icaA*, *icaD*, and *icaB* genes during the adhesion and biofilm formation stages. These observations suggested that the altered *ica* operon expression contributes to the inhibition of biofilm development by BiF2_5K7K at subinhibitory concentrations.

The human β-defensin 3 (hBD‑3) peptide exhibits antibacterial activity against *S. aureus* and effectively inhibits its biofilm formation. This biofilm inhibition is attributed to the impact of hBD-3 on genes related to biofilm development. At a subinhibitory concentration (8 µg/mL), hBD-3 demonstrates the ability to inhibit biofilm development by modulating the expression of the *ica* operon. A substantial upregulation of the *ica* operon was observed when compared to the control [53]. Moreover, the increased biofilm gene expression of *S. epidermidis* may be a protective mechanism against the presence of the peptide, thereby preserving cell viability. This finding is supported by previous studies which show that in response to environmental stress, treatment with rifampicin, clindamycin, gentamicin, and vancomycin alone or in combination affect the expression of the *icaA* and *rsbU* genes [28, 53].

During the development of biofilm, the quorum-sensing system is the regulatory system that strongly impacts biofilm development [8]. One of the most studied quorum-sensing systems in *S. epidermidis* is the accessory gene regulator (*agr*) system. The *agr* quorum-sensing system is comprised of two different transcription units, identified as RNAII and RNAIII. The RNAII locus consists of four genes: *agrB*, *agrD*, *agrC*, and *agrA*. The presence of BiF2_5K7K may have reduced the levels of *agrD* and *agrB* gene expression, causing interference with the *agr* quorum-sensing system. Downregulation of the *agrD* and *agrB* genes leads to inhibition of RNAIII, which is the main effector molecule of the *agr* quorum-sensing system. The BCp12 peptide, a novel peptide derived from buffalo casein hydrolysate, demonstrates a comparable capability to BiF2_5K7K by effectively preventing the formation of *S. aureus* biofilm at both the MIC and sub-MIC concentrations. This inhibition occurs through the downregulation of *agrA*, *agrB*, *agrC*, and *psmβ* gene expressions, resulting in interference of the *agr* quorum-sensing system [54]. Based on our findings, BiF2_5K7K inhibited *S. epidermidis* biofilm formation by inducing environmental stress, and thereby modifying the expression of polysaccharide production and downregulating the *agr* quorum-sensing system. The *ica* operon in *S. epidermidis* plays an important role in device-related infection. Therefore, altering this operon could be a crucial step in combating bacterial biofilm infections [52]. The downregulation of genes in the *agr* quorum-sensing system implied a disruption in bacterial communication mechanisms, suggested a potential to inhibit biofilm formation, which is a crucial step in combating bacterial biofilm infection [8]. By interfering with these systems, BiF2_5K7K could prevent the ability of bacteria to establish biofilm and enhance the efficacy of conventional antibiotic treatments when used in combination.

Amphipathicity, often referred to as peptide helicity [55], plays a pivotal role in determining how peptides interact with bacterial membranes. Peptide helicity has been demonstrated by examining the secondary structural changes induced by electrostatic interactions between the peptide and the bacterial membrane [45]. In this study, BiF2_5K7K exhibited an amphipathic α-helical conformation. The circular dichroism spectra further validated the secondary structure of BiF2_5K7K under a membrane-mimicking condition. While the structure of BiF2_5K7K remained disordered in aqueous solution, an α-helical amphipathic structure was observed under differing membrane-mimicking environments, suggesting its antibiofilm and antimicrobial activities.

Eliminating mature biofilms poses a considerable challenge in biofilm treatment, as extracellular matrix may constitute up to 90% of an established biofilm, encasing the bacteria and delaying or preventing antibiofilm agent penetration into the biofilm structure [10, 16]. Interestingly, previous research has highlighted the unique potential of peptides in combating biofilms. Such efficacy can be attributed to the amphipathic properties and flexible molecular structures of the peptides, enabling them to readily penetrate biofilm structures. This enhanced ability to infiltrate the extracellular matrix is a crucial factor contributing to their efficacy in preventing and disrupting biofilms. In this study, BiF2_5K7K demonstrated the ability to eradicate mature *S. epidermidis* biofilms and this was likely due to their amphipathic properties and flexible structures, allowing free biofilm penetration [56]. However, higher concentrations of the peptide were needed for this eradication compared to that required to prevent initial development. This suggested that function of the peptide may be limited by the presence of extracellular matrix.

Urinary catheters represent one of the commonly used devices in healthcare settings. However, their long-term use is limited by the risk of catheter-associated urinary tract infections (CAUTI) which may cause up to 23% of infections in intensive care units (ICUs) and 40% of hospital-related infections [57, 58]. Most (70–80%) of these infections occur owing to bacterial contamination either before or during insertion [59], and often develop highly drug-resistant biofilm. Coating catheters with antibiofilm peptides represents an attractive approach to limiting this problem. These peptides have been demonstrated to have potent antibiofilm and broad-spectrum antibacterial activities while causing minimal toxicity and inducing resistance. As a preliminary test, BiF2_5K7K was immobilized onto a catheter via physical adsorption. Approximately 1% (0.4 to 1.6%) of BiF2_5K7K was distributed along the catheter, indicating that physical adsorption may not be the optimal method for coating catheters. Despite this, the results indicated that a BiF2_5K7K-coated catheter can inhibit *S. epidermidis* biofilm formation on the catheter surface as well as in the surrounding area, though it cannot prevent the attachment of sessile and planktonic bacteria. Previous research suggested that peptide coatings typically exhibit less antimicrobial activity than their soluble forms, maybe attributed to the non-specific nature of immobilization techniques [59]. The biofilm inhibitory activity of the BiF2_5K7K-coated catheter was found to be dependent on the concentration of the coating. Higher concentrations exhibited greater efficacy in inhibiting biofilm formation on both the catheter surface and its surrounding areas. However, no correlation was observed between the ability to prevent bacterial attachment on the coated catheter and that in the surrounding area. These findings may be attributed to the concentration retained within or released from the catheter. To enhance the activity of the coated catheter through adsorption techniques, very high concentrations may be required, or alternative methods for coating peptides such as cysteine-immobilization and polydopamine coating onto the catheter surface may need to be explored. In a study by Mishra et al., Lasio-III was chemically modified with a cysteine residue and subsequently immobilized on silicon surfaces. The resulting cysLasio-III-immobilized catheters demonstrated antimicrobial activity against *E. coli* and *Enterococcus faecalis*, along with antibiofilm properties, showing a reduction of *E. faecalis* and *E. coli* biofilms by 60% and 40%, respectively [60]. Similarly, in another study by Lim et al., the CWR11 tethered onto catheter surfaces using the polydopamine coating technique exhibited no visible colonies when incubated with *E. coli* [61]. In addition, combining antibiofilm peptide with antimicrobial peptides, conventional antibiotics, or compounds with specific functions such as Dnase (to dissolve biofilm matrix) [62], QS inhibitor [10], and disodium ethylenediaminetetraacetic acid (EDTA, a chelating agent) [16] may be preferable to any one approach alone.

In conclusion, BiF2_5K7K was a novel dual-action hybrid peptide with potent antibiofilm activity against a *S. epidermidis* biofilm-forming strain due to interference with gene expression. It demonstrated wide-ranging antibacterial efficacy with low toxicity. This peptide might be further developed as an antibiofilm agent, however further assessment of its stability, efficacy and toxicity in vivo is needed to reach clinical utility.

### Electronic supplementary material

Below is the link to the electronic supplementary material.

Supplementary Material 1

## Data Availability

All of the data produced throughout this research are contained within this article and/or its supplementary information file.
