# Supplementary material for: Dual-function antimicrobial-antibiofilm peptide hybrid to tackle biofilm-forming Staphylococcus epidermidis
Source: Ann Clin Microbiol Antimicrob. 2024 May 16;23:44. doi: 10.1186/s12941-024-00701-7 (PMC11100219; doi:10.1186/s12941-024-00701-7)
Supplement: Supplementary file 1 — Supplementary Material 1 [file 12941_2024_701_MOESM1_ESM.docx]

**Supplementary Information**

Dual-function antimicrobial-antibiofilm peptide hybrid to tackle biofilm-forming *Staphylococcus epidermidis*

Mathira Wongchai^1^, Saharut Wongkaewkhiaw^2^, Sakawrat Kanthawong^3^, Sittiruk Roytrakul4 and Ratchaneewan Aunpad^1,*^

^1^Graduate Program in Biomedical Sciences, Faculty of Allied Health Sciences, Thammasat University, Pathum Thani, Thailand

^2^School of Dentistry, King Mongkut’s Institute of Technology Ladkrabang, Bangkok, Thailand

^3^Department of Microbiology, Faculty of Medicine, Khon Kaen University, Khon Kaen, Thailand

^4^Functional Proteomics Technology Laboratory, National Center for Genetic Engineering and Biotechnology, National Science and Technology Development Agency, Pathum Thani, Thailand

*Corresponding author

Email: aratchan@tu.ac.th; +66829869213


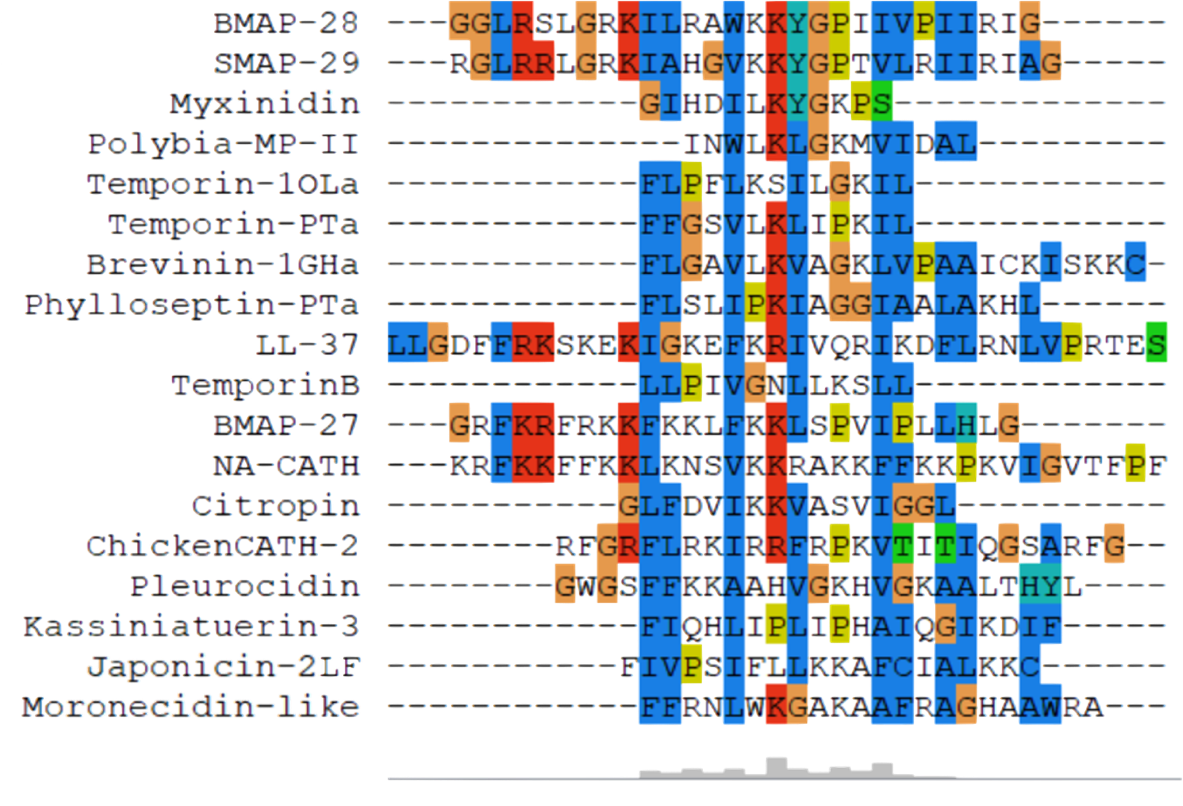


**Fig. S1** Alignments of multiple sequences of 18 α-helical antibiofilm peptides for rational design of novel antibiofilm peptides using ClustalX2.1 program. The hydrophobic residues are shown in blue, positively charged residues in red, negatively charged residues in magenta, and polar in green. Moreover, the special residues glycine, proline and aromatic are shown in orange, yellow and cyan, respectively.


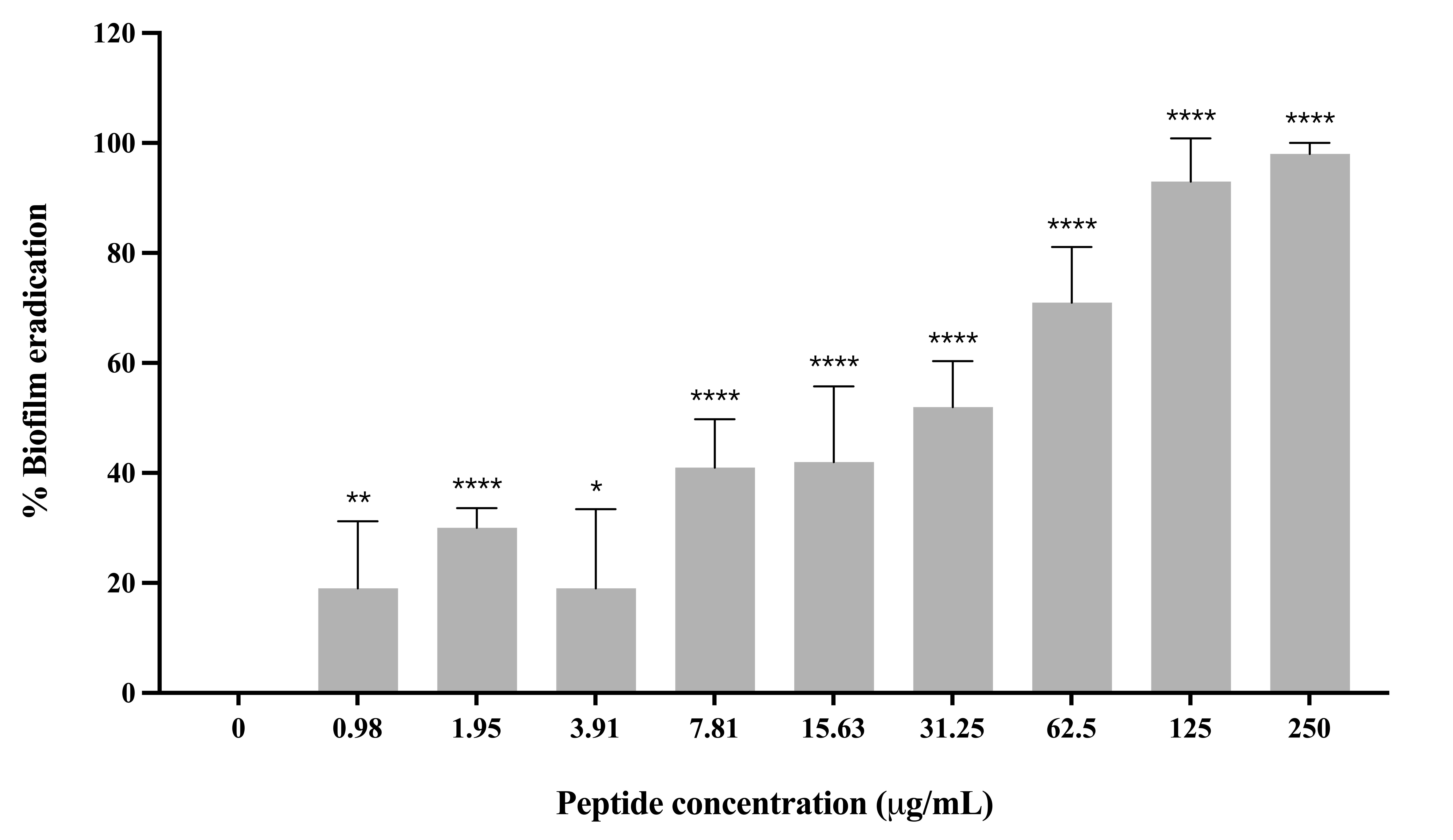


**Fig. S2** Biofilm eradication activity of BiF2_5K7K against *S. epidermidis*. Experiments were conducted in triplicate, and the data are presented as mean ± SD. The statistical analyses were performed by one-way ANOVA at **p*-value < 0.05, ***p*-value < 0.01, ****p*-value < 0.001, and *****p*-value < 0.0001.


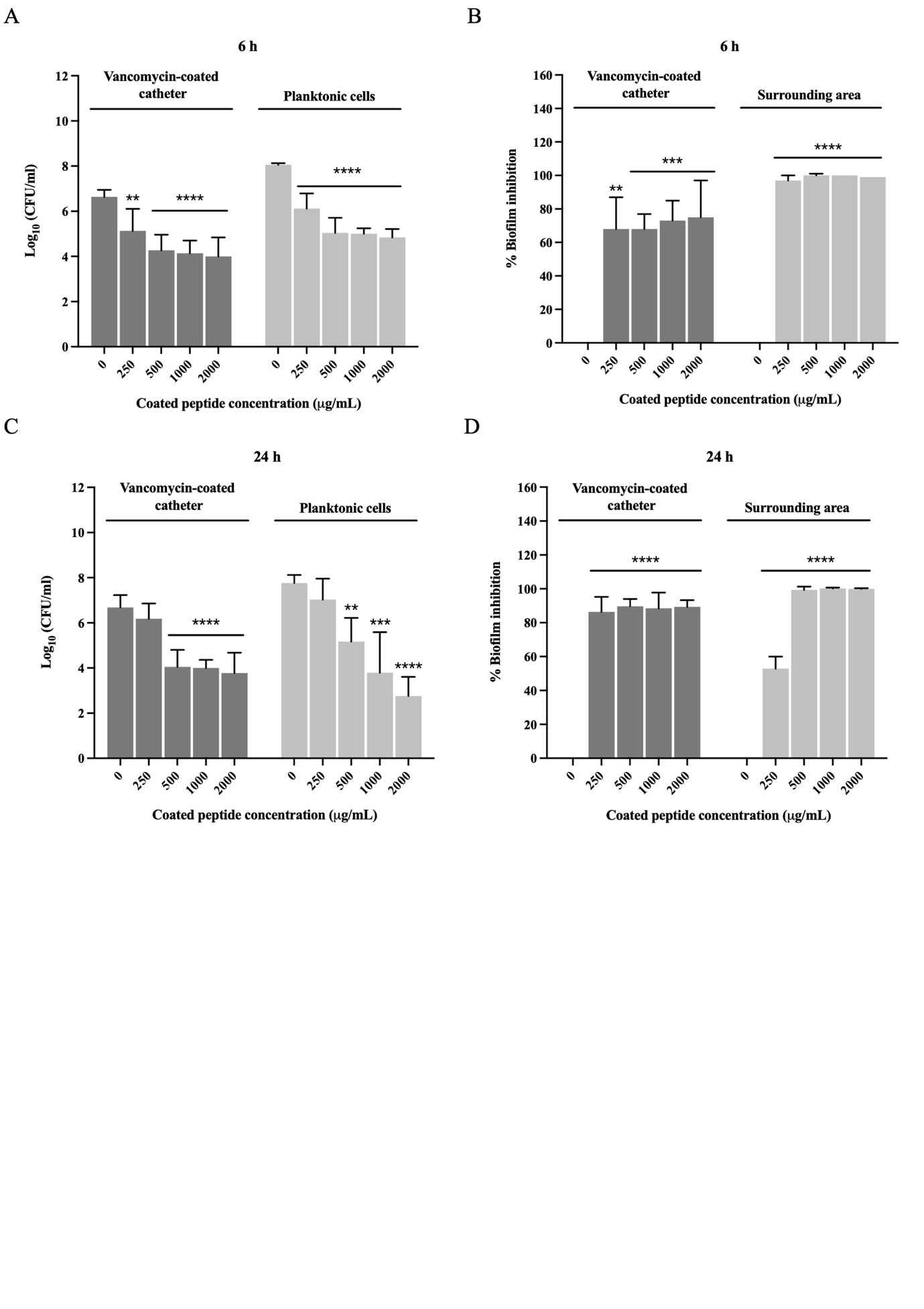


**Fig. S3** Antibiofilm activity of vancomycin on silicone catheters. **A** and **C** Bactericidal effect of vancomycin-coated catheters against sessile and planktonic *S. epidermidis* after 6 and 24 hours of incubation, respectively. **B** and **D** Biofilm inhibitory activity on vancomycin-coated catheters and surrounding area, respectively. The experiments were performed at least in triplicate and the data are presented as mean ± SD. **p<0.01, ***p<0.001, ****p<0.0001 indicate significant differences.

**Table S1**. Gene-specific primers used for real-time RT-PCR.

| Gene | Primer (5’→3’) |
| --- | --- |
| *Polysaccharide intracellular adhesin gene* | |
| *16s rRNA* | F: CCTATAAGACTGGGATAACTTCGGG  R: CTTTGAGTTTCAACCTTGCGGTCG |
| *icaA* | F: ACAGTCGCTACGAAAAGAAA  R: GGAAATGCCATAATGACAAC |
| *icaB* | F: CTGATCAAGAATTTAAATCACAAA  R: AAAGTCCCATAAGCCTGTTT |
| *icaC* | F: TAACTTTAGGCGCATATGTTTT  R: TTCCAGTTAGGCTGGTATTG |
| *icaD* | F: ATGGTCAAGCCCAGACAGAG  R: CGTGTTTTCAACATTTAATGCAA |
| *icaR* | F: TAATCCCGAATTTTTGTGAA  R: AACGCAATAACCTTATTTTCC |
| *Agr quorum-sensing gene* | |
| *agrB* | F: TTCGTTTAGGGATGCAGGTA  R: TACCGTGTGCATGTCTCCTA |
| *agrD* | F: CACTACAATCTTGGAATTTATTGGT  R: CTGGTACTTCTGGTTCGTCAA |
| *agrC* | F: CATCAATATCGCATTCATCG  R: AACCGCGATTATCACCTTTA |
| *agrA* | F: AAGCGGGGAAGTAATTCAGT  R: ACGTTCATCAAGCTGTGCTA |
